# Reconstructing GRACE-type time-variable gravity from the Swarm satellites

**DOI:** 10.1038/s41598-020-80752-w

**Published:** 2021-01-13

**Authors:** H. Maja P. Richter, Christina Lück, Anna Klos, Michael G. Sideris, Elena Rangelova, Jürgen Kusche

**Affiliations:** 1 Institute of Geodesy and Geoinformation, University of Bonn, Bonn, Germany; 2 Faculty of Civil Engineering and Geodesy, Military University of Technology, Warsaw, Poland; 3 Department of Geomatics Engineering, Schulich School of Engineering, University of Calgary, Calgary, Canada

**Keywords:** Climate change, Hydrology

## Abstract

The Gravity Recovery and Climate Experiment (GRACE) mission has enabled mass changes and transports in the hydrosphere, cryosphere and oceans to be quantified with unprecedented resolution. However, while this legacy is currently being continued with the GRACE Follow-On (GRACE-FO) mission there is a gap of 11 months between the end of GRACE and the start of GRACE-FO which must be addressed. Here we bridge the gap by combining time-variable, low-resolution gravity models derived from European Space Agency’s Swarm satellites with the dominating spatial modes of mass variability obtained from GRACE. We show that the noise inherent in unconstrained Swarm gravity solutions is greatly reduced, that basin averages can have root mean square errors reduced to the order of $\text {cm}$ of equivalent water height, and that useful information can be retrieved for basins as small as $1000 \times 1000\,\hbox {km}$. It is found that Swarm data contains sufficient information to inform the leading three global mass modes found in GRACE at the least. By comparing monthly reconstructed maps to GRACE data from December 2013 to June 2017, we suggest the uncertainty of these maps to be $2{-}3\,\text {cm}$ of equivalent water height.

## Introduction

For nearly 15 years the Gravity Recovery and Climate Experiment (GRACE) mission allowed us to construct monthly maps of changes in the Earth’s gravitational field (and thus mass distribution) with unprecedented accuracy. This data has been used to study glacier and ice sheet mass imbalance^1,2^, hydrological change^3,4^ (including floods and droughts^5–7^) ocean mass change^8,9^, the solid Earth’s response to post-glacial unloading^10–12^, and the mechanisms of large earthquakes^13,14^. The GRACE mission surpassed its planned duration three times over, with its mass change times series being brought to a close in June 2017, and the satellites being decommissioned later that same year. The GRACE successor mission, GRACE Follow-On (GRACE-FO), was then successfully launched in May 2018, with gravity field models being produced from June 2018 onwards. As a result, there is a gap of 11 months between these missions.

For science applications that require a continuous time series, at least four methods exist that can be used to bridge this gap: (1) GRACE data are simply extrapolated, e.g., by estimating a model consisting of seasonal, trend and acceleration terms based on monthly GRACE level-2 data. This may be combined with a monthly climatology (i.e. averaging all instances of a specific month within the GRACE record). However, this approach is basically equivalent to predicting unobserved gravity models using only knowledge of the past, thus should be used with caution. (2) GRACE data are reconstructed based on data assimilation^15,16^ or other elaborate statistical^17^ or machine learning^18^ methods that explore the correlation of total water storage to observable fields such as precipitation and land or sea surface temperature. Some studies (e.g. Ref.^19^) have developed pre-GRACE era reconstructions in this way, but so far this method has been limited to specific compartments (e.g. only hydrological storage variations) and often to basin averages or continental areas. (3) GRACE-type surface mass fields are reconstructed from the time-variable deformation of the planet as measured by Global Navigation Satellite System (GNSS) networks, and then inverted through a loading theory (see, e.g., Refs.^20–22^). However, the required networks are sparse in many regions of the world, and GNSS time series for individual stations include many non-loading signals and technique-related errors which are difficult to separate^23–25^. (4) Data from other sources such as satellite laser ranging (SLR) or from the European Space Agency (ESA) Swarm 3-satellite formation are used to reconstruct low-degree models of the gravity field^26–28^.

The last method would provide an independent and direct approach to gravity field and mass change estimation, and SLR-, Swarm- and GRACE-results can be compared during the overlap period of December 2013 to June 2017. However, the high accuracy of the GRACE solutions is due to the ultra-precise inter-satellite ranging system, while with SLR and Swarm, the gravity field solutions can only be retrieved from the tracking of the spacecraft orbit perturbations, inevitably resulting in lower spatial resolution. In the case of Swarm, the monthly gravity fields are limited to spherical harmonic degree and order (d/o) of about 12, corresponding to a spatial resolution of 3000–4000 km.

Previous GRACE and modelling studies have shown that the observed mass change in the cryosphere, hydrosphere and oceans can be, to a large extent, explained through a number of modes that represent the temporal evolution of spatially coherent patterns^29–31^. This fact has been exploited in signal separation by Refs. ^3,32–34^, who identified ENSO events in terrestrial water storage changes. Reference^26^ consequently employed it for reconstructing pre-GRACE ice mass changes by combining SLR and Doppler Orbitography and Radiopositioning Integrated by Satellite (DORIS) with spatial modes from GRACE.

Reference^27^ provided a comprehensive description of the quality of the gravity recovery approach using Swarm, proving that its accuracy is comparable to Swarm gravity models delivered by others. References^27^ and^35^ both compare the Swarm solutions of different institutes [Astronomical Institute of the University of Bern (AIUB), Astronomical Institute of the Czech Academy of Science (ASU), Institute of Geodesy (IfG) of the Graz Universtity of Technology, Institute of Geodesy and Geoinformation (IGG) of the University of Bonn, Ohio State University (OSU)] and conclude that spherical harmonic degrees higher than 12 should not be used to derive geophysical signals. In this work, we use the approach of Ref.^27^ and focus on improving the spatial resolution of the monthly Swarm models (which are derived up to d/o 40) while ingesting some a-priori information from the GRACE period. Here, Swarm GNSS-tracking data during December 2013 to December 2018 have been used to retrieve the temporal evolution of three leading spatial modes, which were derived from GRACE during April 2002 to June 2017. One has to keep in mind that the gravity fields reconstructed in this way are spatially constrained by the number of the leading GRACE modes. We evaluate the impact of these constraints by comparing the monthly Swarm-only solutions and the reconstructed Swarm solutions to the monthly GRACE solutions during the GRACE-Swarm overlap period. The comparisons are conducted each for a maximum d/o of 12 and 40. In this way, we investigate both the common Swarm resolution and also the actual available resolution, which mostly contains noise and is thus in general not used (see *Swarm Data* in the Supplement, Refs.^27^ and^35^). We furthermore validate our results by comparisons to vertical deformations from Global Positioning System (GPS) and through evaluating range residuals from SLR.

This article is organized as follows: in the following section we describe the time-variable Swarm gravity fields alongside an error assessment that we obtain from projecting monthly Swarm-only gravity fields^27^ onto the spatial modes from GRACE. We consider two variants of the reconstruction approach: in the first one, the entire observed mass change signal is decomposed and projected; in the second variant, a deterministic signal model is first estimated from the GRACE data, then removed prior to decomposition and ingesting Swarm data, before subsequently being restored. This can be viewed as combining the GRACE interpolation approach with the Swarm reconstruction method. We furthermore show comparisons to GPS data and residuals from SLR data. This is followed by a conclusion and the explanation of our methods.

## Time-variable gravity from Swarm

### Monthly Swarm reconstructions

**Figure 1:**
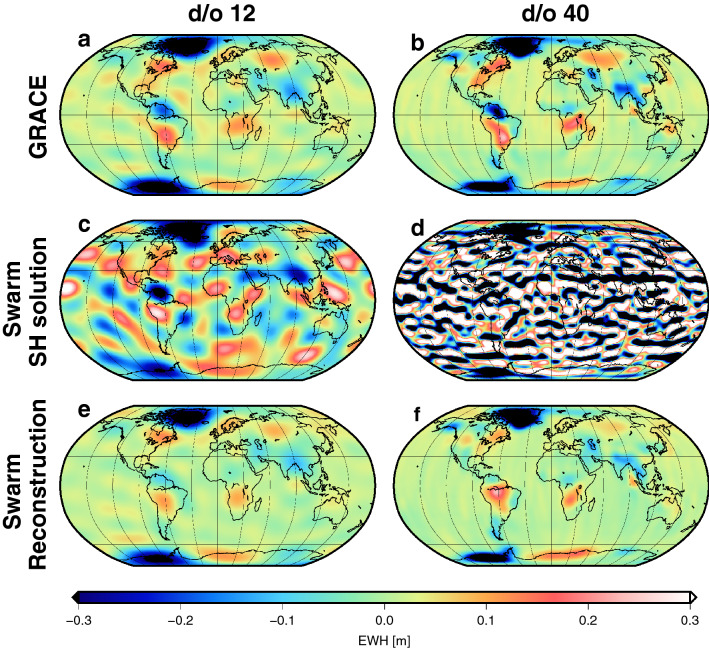
March 2016 e.w.h. surface mass change map. Left: d/o 12, right: d/o 40. (**a**,**b**) From original GRACE gravity field. (**c**,**d**) From Swarm-only gravity field^27^. (**e**,**f**) Reconstruction from monthly Swarm solutions using three GRACE EOFs.

We have reconstructed monthly Swarm geopotential solutions and surface mass change maps (measured in e.w.h., equivalent water height) from December 2013 to December 2018 using principal component analysis (PCA^36^). PCA decomposes the time series of GRACE e.w.h. maps into orthogonal spatial patterns, each scaled by uncorrelated time series. We utilize the GRACE-derived patterns in order to represent two variants of the monthly Swarm solutions, referred to as (1) “Swarm reconstruction” and (2) “${\text {Swarm reconstruction}}_{\text {residual}}$” [see explanation in Methods; if not mentioned explicitly, the results are shown for the Swarm reconstruction (1)]. As an example, Fig. 1 shows monthly surface mass change maps for March 2016 from GRACE (top), Swarm-only (center), and Swarm projected to the leading three GRACE-modes (bottom), each for d/o 12 (left column) and for d/o 40 (right column). The low-degree solution shows that Swarm-only gravity recovery (Fig. 1c) is able to capture some major patterns of the more precise GRACE solution (Fig. 1a), but at monthly time-scale noise is ubiquitous. The Swarm reconstruction (Fig. 1e) constrained by the GRACE spatial patterns is closer to the GRACE monthly solution, with a global root mean square error (RMSE) of $0.022\,\hbox {m}$ compared to the Swarm-only solution (RMSE $0.092\,\hbox {m}$). The improvement through the reconstruction approach is even more striking when we compare d/o 40 solutions (Fig. 1b,d,f); the Swarm-only solution contains mostly noise (RMSE $0.39\,\hbox {m}$), while the reconstructed field is very similar to the GRACE solution (RMSE $0.035\,\hbox {m}$). For the selected month, the ${\text {Swarm reconstruction}}_{\text {residual}}$ variant shows a slightly larger RMSE of $0.035\,\hbox {m}$ for d/o 12 and a slightly lower RMSE of $0.030\,\hbox {m}$ for d/o 40, as compared to the Swarm reconstruction, based on the full signal.

**Figure 2:**
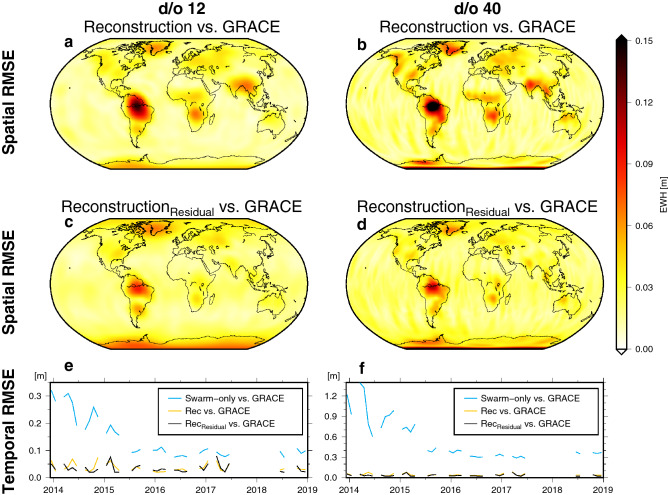
Spatial (**a**–**d**) and temporal (**e**, **f**) RMS errors of the Swarm ${\text {reconstructed}}_{({\text {residual}})}$ solution with respect to GRACE.

Figure 2 provides an assessment of the errors of the method proposed here compared to GRACE. The maps in Fig. 2a,b visualize the spatial error of the Swarm reconstruction, while Fig. 2c,d depict the error of the ${\text {reconstructed}}_{\text {residual}}$ solution. Errors are below 10 cm for most regions. Largest RMS errors are found in the Amazon basin, where the large hydrological signal can neither be fully captured by Swarm nor fully represented in three modes. Moreover, climate phenomena like El Niño and La Niña lead to propagating mass change that also cannot be explained with only three modes (e.g., drought conditions in Northern South America towards the end of 2015). The degree and order 12 and 40 error maps appear broadly similar, with larger errors for higher degrees. Furthermore, the ${\text {Swarm reconstruction}}_{\text {residual}}$ variant is comparable to the Swarm reconstruction solution, with, in general, slightly smaller RMSE values over land and slightly larger RMSE values over the ocean and Antarctica. When we assess the global mean RMSE as a function of time (Fig. 2e,f) the d/o 40 solutions again exhibit larger errors, in particular the Swarm-only solution (0.29–$1.39\,\hbox {m}$). The reconstruction method reduces the errors significantly (0.025–$0.085\,\hbox {m}$). Both reconstruction variants show a similar performance with the ${\text {Swarm reconstruction}}_{\text {residual}}$ variant performing better towards the beginning. The large errors observed at the start of the Swarm mission can be attributed mainly to higher ionospheric activity during 2014 and 2015. This affected the performance of Swarm GNSS receivers^37^, an effect which was mitigated through receiver updates in the early mission phase^38^. The reconstruction is much less affected by ionospheric disturbances as compared to the monthly Swarm-only solutions.

### Basin averages

**Figure 3:**
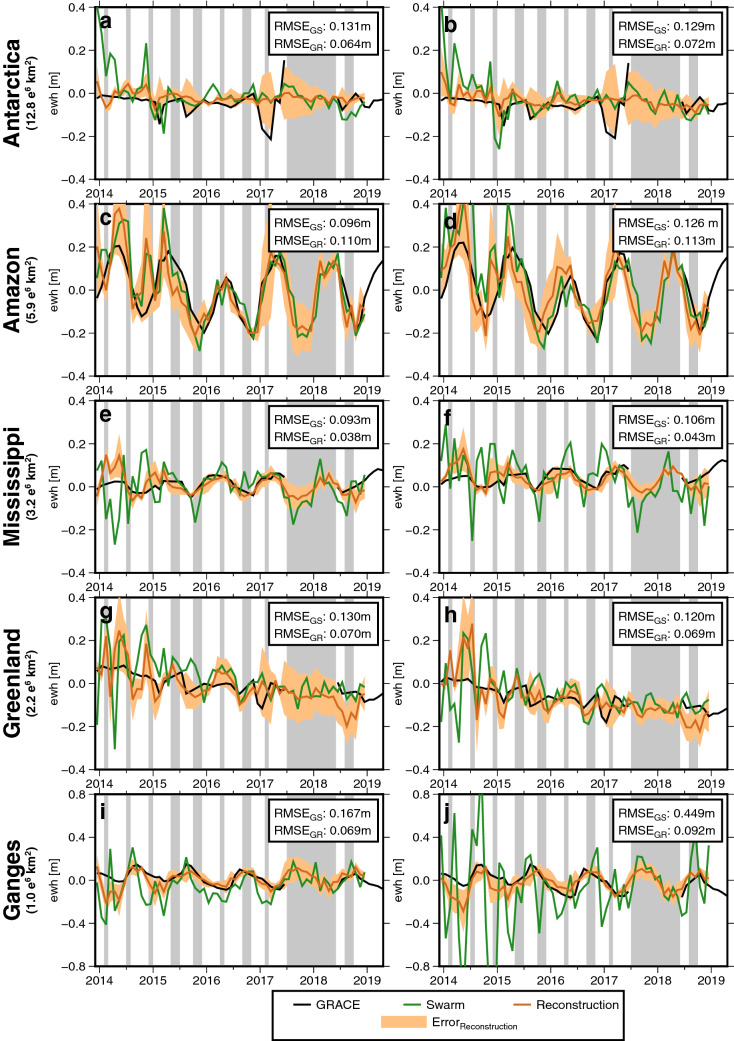
Basin averages for different regions expressed in meter of e.w.h. The original GRACE solution, the original Swarm-only solution, as well as the reconstructed gravity field are shown. Left: d/o 12, right: d/o 40. The error of the reconstruction is indicated in yellow as computed by Eq. (6). The grey bars in each plot indicate months with no GRACE and GRACE-FO data. ${\text {RMSE}}_{\text {GS}}$ indicates the RMSE of Swarm w.r.t. GRACE, while ${\text {RMSE}}_{\text {GR}}$ refers to the RMSE of the reconstruction approach w.r.t. GRACE. All RMSE values are related to the overlapping GRACE and Swarm period (December 2013–June 2017).

**Figure 4:**
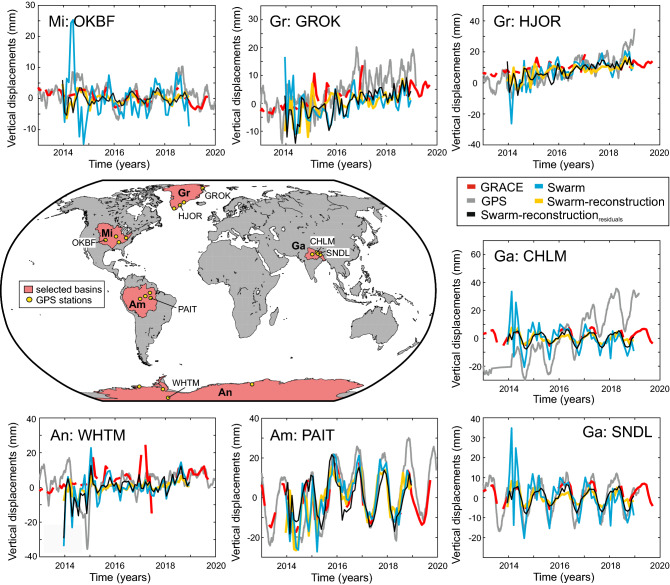
Study regions. An: Antarctica, Am: Amazon, Mi: Mississippi, Gr: Greenland, Ga: Ganges. For each basin, 4 GPS stations were randomly chosen and analyzed. Time series plots present the vertical displacements for individual stations estimated from: Swarm reconstruction (yellow), Swarm ${\text {reconstruction}}_{\text {residual}}$ (black), Swarm-only (blue), GRACE (red) and GPS (gray). Remaining stations are included in the supplementary materials. Stations shown at the charts are marked.

In order to assess the impact of the new approach for typical regional applications, we here compare area-mean mass changes (in terms of e.w.h.) for basins of various size. Figure 3 shows basin averages (for the locations, see Fig. 4) for the Antarctic ice sheet, the Amazon basin, the Mississippi basin, the Greenland ice sheet and the Ganges basin derived from GRACE, Swarm-only and the Swarm reconstruction for d/o 12 and 40. Tables 1 and 2 give an overview of trend and RMSE estimates and also include a six-parameter GRACE model (constant, trend, and annual and semiannual terms).

**Table 1:** Trends (${\hbox {myr}}^{-1}$), RMSE (${\text {m}}$) and RMS (${\text {m}}$) for the d/o 12 solutions in our study regions. The following cases are investigated: (1) the monthly ITSG-Grace2018 solution (${\text {GRACE}}_{\text {real}}$), (2) the 6-parameter signal model (constant+trend+(semi-)annual) of the monthly ITSG-Grace2018 solution (${\text {GRACE}}_{\text {signal}}$), (3) the monthly Swarm-only solution, (4) the Swarm-reconstructed solution (Swarm-rec), (5) the Swarm-reconstructed solution, which is computed on the basis of Swarm and GRACE residuals (${\text {Swarm-rec}}_{\text {residual}}$). RMSE values are all computed w.r.t. ${\text {GRACE}}_{\text {real}}$.

	Antarctica		Amazon		Mississippi		Greenland		Ganges	
Trend ${\text {GRACE}}_{\text {real}}$	$-$ 0.01^a^	($-$ 0.01^b^)	$-$ 0.04	($-$ 0.02)	0.01	(0.01)	$-$ 0.04	($-$ 0.03)	$-$ 0.02	($-$ 0.02)
Trend ${\text {GRACE}}_{\text {signal}}$	$-$ 0.01	($-$0.01)	0.00	(0.00)	0.00	(0.00)	$-$ 0.05	($-$0.05)	0.00	(0.00)
Trend Swarm-only	$-$ 0.05	(0.03)	$-$ 0.06	($-$ 0.02)	0.01	(0.01)	$-$ 0.03	($-$ 0.04)	$-$ 0.01	($-$ 0.02)
Trend Swarm-rec	$-$ 0.01	(0.00)	$-$ 0.07	(0.00)	$-$ 0.01	(0.00)	$-$ 0.04	($-$ 0.01)	0.01	(0.00)
Trend ${\text {Swarm-rec}}_{\text {residual}}$	$-$ 0.02	(0.02)	0.00	(0.02)	0.00	($-$ 0.01)	$-$ 0.05	($-$ 0.05)	$-$ 0.01	(0.00)
RMSE ${\text {GRACE}}_{\text {signal}}$ versus ${\text {GRACE}}_{\text {real}}$	0.07	(0.08)	0.07	(0.08)	0.02	(0.02)	0.06	(0.06)	0.03	(0.03)
RMSE Swarm-only versus ${\text {GRACE}}_{\text {real}}$	0.13	(0.08)	0.10	(0.08)	0.09	(0.04)	0.13	(0.08)	0.17	(0.13)
RMSE Swarm-rec versus ${\text {GRACE}}_{\text {real}}$	0.06	(0.07)	0.11	(0.07)	0.04	(0.03)	0.07	(0.05)	0.07	(0.06)
RMSE ${\text {Swarm-rec}}_{\text {residual}}$ versus ${\text {GRACE}}_{\text {real}}$	0.08	(0.07)	0.07	(0.08)	0.04	(0.03)	0.08	(0.06)	0.04	(0.03)
RMS(Swarm-only-Swarm-rec)^c^	0.04		0.06		0.06		0.07		0.09	
RMS(Swarm-only-${\text {Swarm-rec}}_{\text {residual}}$)^c^	0.02		0.06		0.08		0.06		0.08	

Considered time periods:

^a^2013-12 to 2017-06: overlapping GRACE/Swarm period. This period is shown in the first column of each region in the upper part of the table.

^b^2015-01 to 2017-06: as the Swarm-only solutions suffer from ionospheric disturbances in the early phase of the mission, the values in brackets are related to a shorter period.

^c^2017-07 to 2018-12: in the lower part of the table, the RMS of Swarm-only minus ${\text {Swarm-reconstructed}}_{\text {(residual)}}$ after the end of GRACE is shown.

**Table 2:** Trends (${\hbox {myr}}^{-1}$), RMSE (${\text {m}}$) and RMS (${\text {m}}$) for the d/o 40 solutions in our study regions. The following cases are investigated: (1) the monthly ITSG-Grace2018 solution (${\text {GRACE}}_{\text {real}}$), (2) the 6-parameter signal model (constant+trend+(semi-)annual) of the monthly ITSG-Grace2018 solution (${\text {GRACE}}_{\text {signal}}$), (3) the monthly Swarm-only solution, (4) the Swarm-reconstructed solution (Swarm-rec), (5) the Swarm-reconstructed solution, which is computed on the basis of Swarm and GRACE residuals (${\text {Swarm-rec}}_{\text {residual}}$). RMSE values are all computed w.r.t. ${\text {GRACE}}_{\text {real}}$.

	Antarctica		Amazon		Mississippi		Greenland		Ganges	
Trend ${\text {GRACE}}_{\text {real}}$	$-$ 0.01^a^	($-$ 0.01^b^)	$-$ 0.04	($-$ 0.03)	0.01	(0.01)	$-$ 0.04	($-$ 0.03)	$-$ 0.03	($-$ 0.03)
Trend ${\text {GRACE}}_{\text {signal}}$	$-$ 0.01	($-$ 0.01)	0.00	(0.00)	0.00	(0.00)	$-$ 0.05	($-$ 0.05)	$-$ 0.01	($-$0.01)
Trend Swarm-only	$-$ 0.05	(0.04)	$-$ 0.01	($-$ 0.05)	$-$ 0.03	($-$ 0.01)	$-$ 0.03	($-$ 0.03)	0.02	($-$ 0.01)
Trend Swarm-rec	$-$ 0.02	(0.01)	$-$ 0.06	(0.01)	$-$ 0.01	(0.00)	$-$ 0.05	($-$ 0.01)	0.02	(0.00)
Trend ${\text {Swarm-rec}}_{\text {residual}}$	$-$ 0.01	(0.01)	0.00	(0.01)	0.00	(0.00)	$-$ 0.06	($-$ 0.06)	$-$ 0.01	(0.00)
RMSE ${\text {GRACE}}_{\text {signal}}$ versus ${\text {GRACE}}_{\text {real}}$	0.06	(0.08)	0.08	(0.08)	0.02	(0.02)	0.06	(0.06)	0.04	(0.04)
RMSE Swarm-only versus ${\text {GRACE}}_{\text {real}}$	0.13	(0.08)	0.13	(0.08)	0.11	(0.08)	0.12	(0.08)	0.45	(0.24)
RMSE Swarm-rec versus ${\text {GRACE}}_{\text {real}}$	0.07	(0.08)	0.11	(0.10)	0.04	(0.03)	0.07	(0.05)	0.09	(0.07)
RMSE ${\text {Swarm-rec}}_{\text {residual}}$ versus ${\text {GRACE}}_{\text {real}}$	0.06	(0.07)	0.08	(0.08)	0.02	(0.02)	0.06	(0.04)	0.04	(0.04)
RMS(Swarm-only-Swarm-rec)^c^	0.04		0.08		0.09		0.07		0.18	
RMS(Swarm-only-${\text {Swarm-rec}}_{\text {residual}}$)^c^	0.03		0.06		0.11		0.08		0.18	

Considered time periods:

^a^2013-12 to 2017-06: overlapping GRACE/Swarm period. This period is shown in the first column of each region in the upper part of the table.

^b^2015-01 to 2017-06: as the Swarm-only solutions suffer from ionospheric disturbances in the early phase of the mission, the values in brackets are related to a shorter period.

^c^2017-07 to 2018-12: in the lower part of the table, the RMS of Swarm-only minus ${\text {Swarm-reconstructed}}_{\text {(residual)}}$ after the end of GRACE is shown.

The first observation from Tables 1 and 2 is that, during the Swarm time frame considered here, the monthly GRACE-derived mass anomalies reveal a quite regular behaviour for all regions considered, without larger (e.g. ENSO-related) temporal anomalies and without trend changes or similar obvious interannual changes. The GRACE six-parameter signal model follows the monthly GRACE solution within RMSE 2–8 cm, which is quite moderate when compared to ENSO years. It is thus not surprising that this simple GRACE-based prediction model already provides a good fit, however this is obviously due to the low variability in the considered time frame and therefore cannot invalidate methods that rely on other data such as Swarm.

Trends derived from a 4-year period cannot provide significance for assessing geophysical processes, but they can be compared to each other. We notice, e.g., that for the Amazon basin the GRACE trend within the Swarm period does not fit well to the overall GRACE trend (which one would have to use when relying on GRACE interpolation) while for other regions the overall GRACE trend represents a good predictor for the Swarm period. Again, this is due to only very moderate interannual variability in this timeframe. We notice that trends from a Swarm-only monthly solution are generally larger as compared to the reconstruction approach, which is in line with the methodology. We find that Swarm-only trends appear unrealistically large for the Antarctica and Amazon regions, in particular for d/o 40, while all other approaches including the reconstructions fit to GRACE trends. Results are diverse for the smaller regions.

As for the RMSE with respect to the monthly GRACE solution, the Swarm-monthly solutions generally shows the largest misfit, followed by the Swarm reconstruction and then the ${\text {Swarm reconstruction}}_{\text {residual}}$ with removing-restoring GRACE trends. This is particularly striking for the d/o 40 comparisons where the latter approach indeed outperforms the GRACE 6-parameter model in almost all regions and sub-intervals. This is encouraging, but it may be expected due to the mentioned regular behaviour of mass change during the time frame. It is also worth noting that the Swarm reconstruction without making use of GRACE-trends is much closer to GRACE as compared to the Swarm-monthly result.

For the entire Antarctic ice sheet, GRACE shows a mass loss until mid-2016. Afterwards, the time series shows a slight mass gain until November 2016, followed by a drop of approximately $20\,\hbox {cm}$. This drop is likely an artifact due to the poorer quality of GRACE data caused by the lack of accelerometer data from GRACE B for this period^39^ (in November 2016 the GRACE B accelerometer had been switched off for the first time to save battery power and non-gravitational accelerations measured onboard GRACE A were transplanted to GRACE B). This affects the Antarctic regions in particular, as can be seen in the time series as well as in Figs. S2.1 and S2.2 of the Supplement. For this reason, we only consider the time from 2013-12 to 2016-08 for the Antarctic ice sheet, except if stated otherwise. The d/o 40 GRACE solution that we show here points to a mass loss of $145\,{\hbox {Gtyr}}^{-1}$ (corresponding to ${-}\,0.01 {\hbox {myr}}^{-1}$ mass loss in the Antarctic or $0.4 {\hbox {mmyr}}^{-1}$ global mean sea level rise) within December 2013 to August 2016, which is in the same order of magnitude as the $178{\hbox {Gtyr}}^{-1}$ that Ref.^2^ find from combining GRACE and Cryosat-2 data for a longer time span (January 2011–June 2017). These rates are predominately driven by melting glaciers in West Antarctica and the Antarctic peninsula, while the East Antarctic ice sheet appears more stable. As expected, the monthly Swarm-only solutions suffer from considerable noise, in particular in the beginning. However, for d/o 12, the RMSE between GRACE and Swarm is reduced from $0.14 \, \hbox {m}$ (monthly Swarm-only solution) to $0.05 \, \hbox {m}$ when we use the reconstructed solution (or $0.05 \, \hbox {m}$ for $\text {Swarm reconstruction}_{\text {residual}}$). We find that the trend from the reconstructed Swarm solution (${-}\,0.01 {\hbox {myr}}^{-1}$ for d/o 12) over the time span December 2013–June 2017 is the same as the GRACE trend. After the end of the GRACE lifetime (2017-06 to 2018-12), the Swarm solutions agree with each other by $0.04\,\hbox {m}$ (d/o 12) and $0.035 \, \hbox {m}$ (d/o 40).

For the Amazon basin, GRACE exhibits a large seasonal signal overlaid by a declining trend in the years 2015 and 2016, which reverses in 2017. This is likely due to the 2015–2016 drought caused by a strong El Niño^40^. From comparing GRACE and GRACE-FO in 2017-2019, we conclude that there is no significant trend in the GRACE gap. All Swarm solutions appear to follow GRACE and GRACE-FO well after 2015 (monthly Swarm-only solutions: RMSE $0.08 \, \hbox {m}$ for d/o 12 and d/o 40; reconstruction: RMSE $0.07 \, \hbox {m}$ for d/o 12 and RMSE $0.10 \, \hbox {m}$ for d/o 40; ${\text {reconstruction}}_{\text {residual}}$: RMSE $0.08 \, \hbox {m}$ for d/o 12 and d/o 40), and they confirm the reversal in 2017 as well as a stable development after 2017. Here, the reconstruction does not significantly improve over the monthly Swarm-only solutions, which most likely can be explained by the El Niño not being represented in the first three EOFs. The Swarm-only and Swarm-reconstructed solutions agree with each other at the level of RMSE $0.06 \, \hbox {m}$ for d/o 12 and $0.08 \, \hbox {m}$ for d/o 40 after the end of GRACE (from 2017-06 to 2018-12). They appear able to close the gap between GRACE and GRACE-FO well.

In the Mississippi basin, GRACE detects strong annual water storage changes with a positive trend ($0.01\,{\hbox {myr}}^{-1}$ for d/o 12 and d/o 40). After GRACE, the positive trend is still confirmed by GRACE-FO. Ref.^41^ suggests that the largest mass changes in this heavily managed area are due to soil moisture and groundwater changes including withdrawals, followed by relatively small variations in snow depth. For both d/o 12 and d/o 40, the monthly Swarm-only solution captures the seasonal signal well, but it does not see the positive trend for d/o 40. The noise of the monthly Swarm-only solutions (0.09 and $0.11 \, \hbox {m}$ e.w.h., respectively) can be strongly mitigated via the constrained reconstruction ($0.04 \, \hbox {m}$ for both, d/o 12 and 40) or the ${\text {reconstruction}}_{\text {residual}}$ variant ($0.04 \, \hbox {m}$ for d/o 12 and $0.02 \, \hbox {m}$ for d/o 40). In particular after 2014, the reconstruction represents the GRACE solution very well. After the end of GRACE, we see a continuation of the annual signal with unchanged amplitude.

For the Greenland ice sheet, we find a dominating melt-related trend with seasonal amplitudes of about $1.5 \, \hbox {cm}$. Interestingly, the melting seems to decelerate when we look at GRACE and GRACE-FO from 2017-2019. For the d/o 12 solutions, we find a trend of ${-}\,0.04\,{\hbox {myr}}^{-1}$ for GRACE, ${-}\,0.03\,{\hbox {myr}}^{-1}$ for the monthly Swarm-only solution, ${-}\,0.04\,{\hbox {myr}}^{-1}$ for the reconstruction and ${-}\,0.05\,{\hbox {myr}}^{-1}$ for the ${\text {reconstruction}}_{\text {residual}}$ (for the existing GRACE months from December 2013 to June 2017). After 2016, both Swarm solutions follow the seasonal pattern in GRACE mass solutions quite closely. They do not suggest a further acceleration after GRACE’s lifetime. Gradually, the noise with respect to GRACE is reduced and both Swarm solutions converge towards each other. After the end of GRACE, there is a drop in the Swarm reconstructed solution, while the Swarm-only solution is closer to GRACE. This discrepancy leads to an RMS (between the Swarm-only and the Swam reconstruction solution) of $0.07 \, \hbox {m}$ for both d/o 12 and d/o 40 from 2016-07 to 2018-12. In general, the signal-to-noise ratio in Greenland is quite low, as can be seen in Fig. 3.

The Ganges basin is the smallest study region considered here (approx. $1100 \, \hbox {km} \, \times \, 900 \, \hbox {km}$), even though our results suggest that future work could concentrate on smaller basins. Apart from the seasonal signal in the GRACE data, one can see a slight decline of total water storage in the GRACE-FO era. For example, Ref.^42^ and^43^ describe interannual water storage variability to human water usage, extreme precipitation (floods and droughts) and large-scale ocean-atmospheric interactions such as El Niño and Indian Ocean Dipole, yet these are all dwarfed by the huge seasonal signal. We find that for d/o 12, the monthly Swarm-only solution shows an RMSE of $0.17 \, \hbox {m}$ with respect to GRACE, while the Swarm reconstruction has a much lower RMSE of $0.07 \, \hbox {m}$ and $0.04 \, \hbox {m}$ for the ${\text {reconstruction}}_{\text {residual}}$. The monthly Swarm-only solution for up to d/o 40 exhibits large noise (RMSE $0.45 \, \hbox {m}$) and should not be used, while the reconstruction is closer to GRACE (RMSE $0.09 \, \hbox {m}$). The Swarm-only and Swarm-reconstructed solutions agree at the level of RMSE $0.09 \, \hbox {m}$ for d/o 12 and RMSE $0.18 \, \hbox {m}$ for d/o 40 after the end of GRACE, and they seem to extend the time series without noticeable outliers (except for monthly d/o 40 data).

All trend and RMSE values are summarized in Tables 1 and 2. A common approach to bridge the GRACE gaps is to interpolate or extrapolate previous and subsequent GRACE solutions. However, it can be argued that the skill of such simple extrapolation approaches inevitably depends on how “regular” the true surface mass field evolves in time, and that scientifically meaningful results are often related to anomalous or episodic events. On the basis of comparisons to GRACE-derived basin averages, our findings are that monthly Swarm reconstructions should generally be preferred to a simple six-parameter GRACE model (constant, trend, annual and semiannual terms), although we admit that the simple model, in certain situations, is sufficient. A detailed analysis can be found in Section S4 of the Supplement.

### Validation with GPS

In order to validate the new approach with independent data, we analyze time series from twenty globally distributed GPS (Global Positioning System) sites. Vertical GPS displacements were pre-processed and corrected for non-tidal atmospheric, non-tidal oceanic and post-glacial rebound effects, as described in the *Data* section of the Supplement. Mass redistribution estimates derived from GRACE, Swarm-only, Swarm-reconstructed and ${\text {Swarm-reconstructed}}_{\text {residual}}$ fields of maximum d/o 12 were converted to vertical deformation using elastic loading theory (Fig. 4). Subsequently, we derived linear trends and annual amplitudes from daily GPS displacements, GPS displacements averaged to monthly time scale, and displacements estimated for GRACE, Swarm-only, Swarm-reconstructed and ${\text {Swarm-reconstructed}}_{\text {residual}}$ data.

We find that both Swarm-reconstructed and GRACE-predicted displacements reproduce well inter-annual signals observed by GPS, while the fits for trends and at the annual timescale are moderate. Inter-annual variations are large in particular for the Amazon, as noticed by GPS, and they are captured by GRACE and Swarm-reconstructions. Similarly we find a good agreement at interannual timescales for the Mississippi basin where the hydrological signals are also large. GPS uplift or subsidence trends which may be caused, next to mass loading, by a plethora of other geophysical or anthropogenic effects are difficult to compare. At the annual timescale no clear picture emerges: gravity solutions from the Swarm reconstruction approach seem to outperform monthly Swarm-only solution for some regions with large signal while for others the Swarm-only monthly solutions appear closer to GPS. The ${\text {Swarm-reconstructed}}_{\text {residual}}$ variant, which is applied in remove-restore mode, will provide fits very close to the GRACE-predicted loading and thus performs well where we know, from other studies, that GRACE fits well to GPS; yet neither Swarm-only nor Swarm reconstructed solutions appear to fit GPS worse.

### Validation with satellite laser ranging (SLR)

Another way of validating the global gravity solutions is by assessing how well they would allow the prediction of the orbits of other satellites. Here, we use the Swarm-derived gravity fields for computing post-fit satellite laser ranging (SLR) range residuals to five SLR satellites; Lageos 1 (altitude $5860 \, \hbox {km}$), Lageos 2 (altitude $5620 \, \hbox {km}$), Ajisai (altitude $1490 \, \hbox {km}$), Starlette (altitude $800 \, \hbox {km}{-}1100\,\hbox {km}$) and Stella (altitude $810 \, \hbox {km}$). Lageos data is processed in orbit arcs of 10 days, while for Ajisai, Starlette and Stella 3-day arcs are chosen due to limitations in modeling the atmospheric drag^44^.

**Figure 5:**
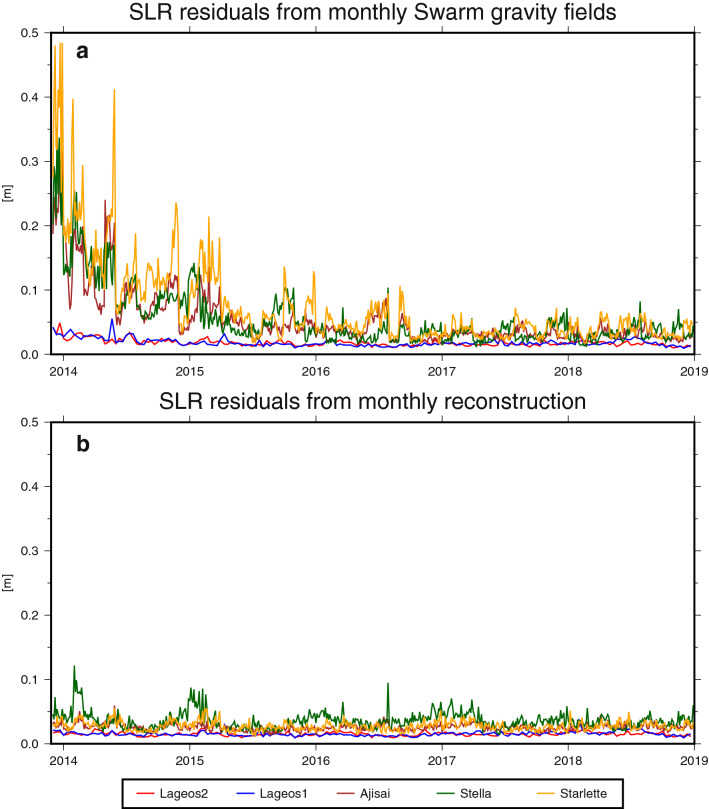
Post-fit range residuals of five SLR satellites. (**a**) Computed with monthly Swarm-only gravity fields. (**b**) Computed with monthly reconstruction.

Figure 5 shows the residuals computed with (a) monthly Swarm-only gravity fields and (b) the reconstruction approach. In both cases, the time-variable gravity fields are truncated at d/o 12 and the same static background model^45^ has been applied for d/o 13–120. As already mentioned, the quality of the monthly Swarm-only solutions is affected by ionospheric disturbances in 2014 and 2015^37^, which can clearly be seen in Fig. 5a. SLR observations form Ajisai, Stella and Starlette do not match the Swarm-only gravity field during the beginning of the Swarm mission, leading to residuals of several decimeters. The SLR residuals reveal (again) that the reconstruction approach does not seem to be affected by the ionospheric disturbances. Starting in 2016, the residuals of both approaches get more similar, but those of the reconstruction are still lower. As Lageos 1/2 fly in a higher orbit than the other three satellites, they are less sensitive to details of the gravity field. Thus, the improvement when using the Swarm reconstruction can mainly been seen when looking at Ajisai, Stella and Starlette. In summary, the analysis of SLR range residuals confirms the capability of the reconstruction approach to improve time-variable gravity fields from Swarm.

## Conclusions

We find that the Swarm time series have the potential to fill the gap between GRACE and GRACE-FO. They follow the GRACE solutions well, but show an elevated level of noise as compared to GRACE, as expected. The two variants of the new reconstruction approach reduce the variance further and improve Swarm results, as we show during the overlap period with GRACE. Our main result is that for the recent period (mid-2017 til mid-2018) which is not covered by GRACE, mass change in all major basins occurs quite regularly compared to earlier GRACE results, as could have been predicted from a climatology. For the Mississippi basin, we find a RMSE of $0.09 \, \hbox {m}$ for the monthly Swarm-only solutions until d/o 12 and $0.04 \, \hbox {m}$ for the reconstruction approach. The improvement becomes even more evident when we consider higher degree solutions: while we suggest that the monthly d/o 40 Swarm-only solutions should rather not be utilized to full resolution, the reconstruction provides reasonable results (RMSE Mississippi: 0.11 and $0.04 \, \hbox {m}$). In general, the RMSE with respect to GRACE appears higher in the early Swarm years because of the strong ionospheric activity that affected Swarm precise orbit determination.

Global reconstructed maps of equivalent water height are much closer to GRACE as compared to monthly Swarm maps. For d/o 40, monthly Swarm-only maps have a global RMSE of $1.39 \, \hbox {m}$ in the beginning of the mission, which decreases to $0.29 \, \hbox {m}$. The reconstructed maps have a lower RMSE of 0.08–$0.02\,\hbox {m}$. The reconstruction approach enables one to derive reasonable monthly maps for d/o 40, something which would not be possible when relying on the Swarm-only monthly solutions.

An independent validation with five SLR satellites confirms that the reconstruction approach can improve time-variable gravity fields from Swarm. Furthermore, comparison to GPS vertical displacements suggests that Swarm-reconstructed deformation fields can cover most of the GPS-observed displacements; relevant for regions where hydrology-induced deformation dominates other effects that GPS is sensitive to.

In this work we have demonstrated a new alternative to simply extrapolating GRACE results in order to fill the GRACE-GRACE-FO mission gap. While this was already possible up to d/o 12 with Swarm gravity data, here we prove that one can achieve higher resolution with a reconstruction approach. Of course this comes at the expense that we have to prescribe GRACE-derived spatial patterns. However, comparing approaches in the overlap period suggests that the new approach clearly outperforms Swarm-only monthly solutions at least for budget studies. We present two variants of the approach: in the first one the entire signal is used in the Principal Component Analysis, leading to a larger flexibility. In the second variant, the PCA is based on GRACE and Swarm residuals, resulting in solutions closer to a 6-parameter GRACE signal model. In other words, this can be viewed as combining GRACE inter-/extrapolation with Swarm analysis of the residual mass fields.

Errors of the reconstruction approach are significantly larger than with GRACE, which was to be expected, but they are considerably smaller than those from Swarm-only data. Our results show that at least for some regions the observed signals are significantly larger than the noise, and scientific interpretation of these signals will provide new insight, in the absence of other methodes.

This work will be valuable for those researchers who have used GRACE results at large-scale or basin-averaged level in the past, for example in order to close the water balance and resolve for evapotranspiration or evaporation minus precipitation^46–49^. Another large field of application is the study of the global sea level budget, where it is of utmost importance to partition the altimetric sea level change ($\approx 3\,{\hbox {mmyr}}^{-1}$) into steric and mass-driven contributions^50^. It would be important here to establish consistency across the GRACE / GRACE-FO era by providing a Swarm estimate. Finally, we believe the newly developed Swarm solutions can provide a blueprint for potential future gaps in the GRACE-FO mission, or between GRACE-FO and a follow-on mission, if required. They could also be applied with other LEOs in order to reconstruct temporal gravity field variability prior to the GRACE mission.

## Methods

### Principal component analysis (PCA)

We pursue two variants using a principal component analysis (PCA^36^) to reconstruct monthly Swarm geopotential solutions: (1) “Swarm reconstruction” and (2) “$\text {Swarm reconstruction}_{\text {residual}}$”. For the Swarm reconstruction (1), we represent the monthly GRACE-derived surface mass changes through a series of *m* mode time series ($a_i(t)$; equals the *i*th column of $\mathbf{A }$) which each scale a corresponding spatial pattern, c.f. Eq. (1). We apply PCA to the monthly e.w.h. grids, following Ref.^29^. PCA decomposes time series of e.w.h. maps, here collected in the $n\times m$ matrix $\mathbf{X }$, with *n* epochs and the *m* grid points,

1 $$\begin{aligned} \mathbf{X }=\mathbf{U }\mathbf{D }\mathbf{V }^T=\mathbf{A }\mathbf{V }^T, \end{aligned}$$

into uncorrelated temporal modes (the principal components, contained in $\mathbf{A }=\mathbf{UD}$, with $\mathbf{U }$ being the $n\times m$ matrix of eigenvectors of $\mathbf{X }^T\mathbf{X }$, and the squared singular values on the diagonal of $m\times m$ matrix $\mathbf{D }$) and orthogonal spatial patterns or Empirical Orthogonal Functions, EOFs (eigenvectors $\mathbf{v }_i$ of $\mathbf{X }\mathbf{X }^T$) contained in the $m\times m$ matrix $\mathbf{V }$. The patterns are typically ordered according to how much variability in the original data they explain, and often few are sufficient to retain the underlying information^26^. Equation (1) can be recast as

2 $$\begin{aligned} \mathbf{x }(t)=\mathbf{v }_1 a_1(t)+\mathbf{v }_2 a_2(t)+\cdots +\mathbf{v }_m a_m(t). \end{aligned}$$

While it is clear that EOF modes and patterns do not necessarily isolate independent physical processes (see discussion in Ref.^32^), the method has been proven capable of effectively rejecting GRACE noise and unphysical stripes^29^ and identifying correlations of total water storage variability with climate modes^34,51^, whose indicators are often defined through PCA of meteorological fields.

Here we suggest to utilize a finite combination of GRACE-derived EOF patterns of surface mass variability in order to represent the monthly Swarm solutions,

3 $$\begin{aligned} \mathbf{l }(t)=\mathbf{v }_1 b_1(t)+\mathbf{v }_2 b_2(t)+\cdots +\mathbf{v }_{{\bar{m}}} b_{{\bar{m}}}(t), \end{aligned}$$

where $\mathbf{l }(t)$ contains all gridded e.w.h. values derived from a given Swarm solution for each monthly epoch *t*. The terms $b_i(t)$ are the Swarm time series that will be multiplied with GRACE patterns and need to be solved for in a least-squares adjustment. It makes sense to resolve only a reduced number ${\bar{m}}$, since Swarm models have less spatial details. Here, we chose ${\bar{m}}=3$, which explains $\sim 90\%$ of the signal. The choice of three EOFs is a result of a global analysis of the RMS of the Swarm reconstruction w.r.t GRACE (as can be seen in Fig. 2 for three EOFs) and a regional analysis of basin averages of our study regions and additional further regions (see Section S5 of the Supplement).

The ${\text {Swarm reconstruction}}_{\text {residual}}$ variant (2) works analogously, but is is based on GRACE and Swarm residuals. The first step in this approach is to compute a 6-parameter model (constant, trend, annual and semiannual terms) from the whole GRACE(-FO) and Swarm period, respectively and subtract it from the original data. The reconstruction, as explained above, is then computed with the residual data. Finally, the GRACE 6-parameter model is added back to the solution, which consequently consists of the main GRACE signal with added deviations computed from GRACE and Swarm residual data. Here, 3 EOFs have also proven to deliver best results w.r.t. original GRACE data.

**Figure 6:**
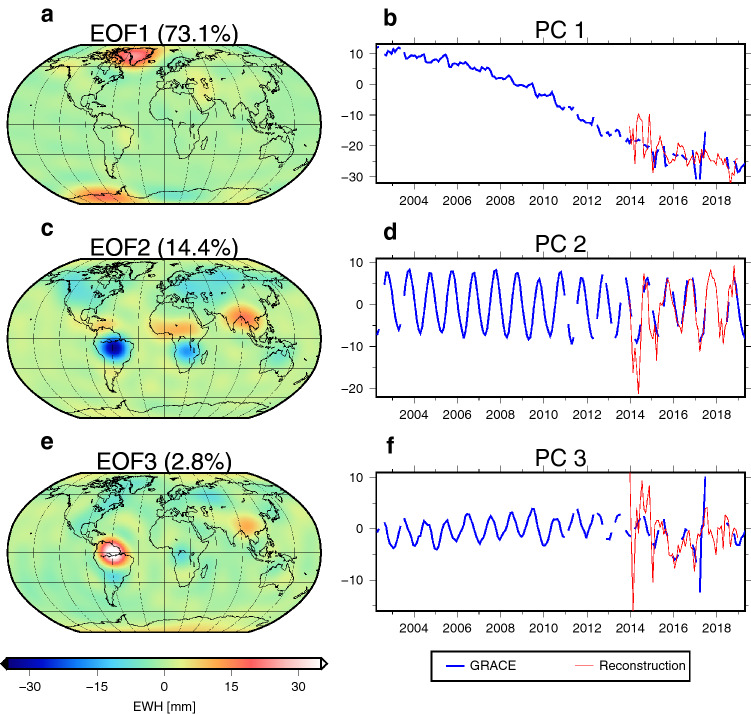
First three dominant orthogonal patterns from monthly GRACE data (left), and corresponding temporal modes derived from GRACE and from Swarm (right).

Figure 6 shows the first three dominating spatial patterns and corresponding modes from GRACE until d/o 12. The first mode explains $73.1 \%$ of the surface mass variability and represents known long-term trends, such as ice-mass loss over Greenland, Antarctica, and Alaska glaciers due to warming oceans (as already shown by e.g. Refs.^32^ or^26^). The corresponding PC shows an acceleration until 2015, followed by a deceleration towards the end of GRACE, which continues for GRACE-FO. It should be mentioned that the months from November 2016 to June 2017 are of minor quality, due to missing accelerometer data as can be seen in Fig. S2.1 and S2.2 of the Supplement^39^. The second mode ($14.4 \%$) captures the large seasonal mass changes in the terrestrial hydrological cycle, while the third mode, also seasonal, contains only $2.8\%$ of the variability.

Figure 6 also shows the corresponding temporal modes derived from Swarm (in red), which capture the main signals quite well. An acceleration signal is visible in the first mode which results from the combined acceleration of mass loss over the large ice shields^52^; the Swarm mode is noisier but captures the trend well. It furthermore confirms the deceleration signal after 2015. The second and the third mode in GRACE describe the annual mass redistribution amplitude and its phase, and they represent inter-annual variability. Apart from 2014, Swarm reproduces those modes well.

### Error budget

In this section, we assess the uncertainty of the Swarm reconstruction. The commission error considers the error between the Swarm reconstruction and the GRACE solution (considered as the truth in the overlap period), if we use the same number of EOFs for both:

4 $$\begin{aligned} \mathbf{e }_c(t)=\left[ \mathbf{v }_1 a_1(t)+\mathbf{v }_2 a_2(t)+\cdots +\mathbf{v }_{{\bar{m}}} a_{{\bar{m}}}(t) \right] -\left[ \mathbf{v }_1 b_1(t)+\mathbf{v }_2 b_2(t)+\cdots +\mathbf{v }_{{\bar{m}}} b_{{\bar{m}}}(t) \right] . \end{aligned}$$

The omission error takes into account that we are not able to reconstruct the full number of EOFs for the reconstruction. We can assess this error by assembling the GRACE signal starting with EOF ${\bar{m}}+1$ until EOF *m*:

5 $$\begin{aligned} \mathbf{e }_o(t)=\mathbf{v }_{{\bar{m}}+1} a_{{\bar{m}}+1}(t)+\mathbf{v }_{{\bar{m}}+2} a_{{\bar{m}}+2}(t)+\cdots +\mathbf{v }_m a_m(t). \end{aligned}$$

We describe the total error $\mathbf{e }(t)$ as

6 $$\begin{aligned} \mathbf{e }(t)=\sqrt{\mathbf{e }_c^2(t)+\mathbf{e }_o^2(t)}. \end{aligned}$$

## Supplementary information

Supplementary material 1
