# Supplementary material for: Reconstructing GRACE-type time-variable gravity from the Swarm satellites
Source: Sci Rep. 2021 Jan 13;11:1117. doi: 10.1038/s41598-020-80752-w (PMC7806766; doi:10.1038/s41598-020-80752-w)
Supplement: Supplementary file 1 — Supplementary material 1 [file 41598_2020_80752_MOESM1_ESM.pdf]

Reconstructing GRACE-type time-variable gravity from the Swarm satellites

H. Maja P. Richter[1], **Christina Lück**[1,*], **Anna Klos**[2], **Michael G. Sideris**[3], **Elena Rangelova**[3], **and Jürgen Kusche**[1]

[1]Institute of Geodesy and Geoinformation, University of Bonn, Bonn, Germany

[2]Faculty of Civil Engineering and Geodesy, Military University of Technology, Warsaw, Poland

[3]Department of Geomatics Engineering, Schulich School of Engineering, University of Calgary, Calgary, Canada

[*]lueck@geod.uni-bonn.de

Supplementary Information

S1 Data

Swarm Data

The prime data source for retrieving time-variable gravity fields from Swarm are precise satellite ephemerides, derived from GNSS-tracking in a so-called kinematic processing where no a-priori gravity field is included. Two versions of Swarm gravity fields have been computed at the Institute for Geodesy and Geoinformation (IGG), University of Bonn, from kinematic orbits from ESA (12-2013 to 03-2015 and 01-2017 to 12-2018) and from Institut für Erdmessung (IfE), University of Hannover (04-2015 to 12-2017)[1]: (1) independent monthly solutions are derived and (2) a model for mean, linear and annual variability is fitted to each spherical harmonic coefficient. For both versions, the integral equation approach with short arcs is used for gravity field retrieval. For more information on this method and the processing at IGG, we refer the reader to ref.[2] and ref.[1]. The Swarm gravity fields have been solved complete to d/o 40, but due to excessive noise in higher harmonics one usually truncates at about d/o 12. Here we only use the monthly Swarm solutions (version (1) mentioned above). Furthermore, to be consistent with GRACE, the same background models (e.g. tides and dealiasing product) as with GRACE RL06 have been applied in the analysis[3].

As the Swarm satellites are not equipped with a precise inter-satellite ranging instrument, derived gravity models inevitably have lower resolution compared to GRACE. Even though different institues derive their monthly time-variable Swarm gravity fields up to different maximum degrees, only degrees up to 12 (corresponding to a spatial resolution of 3000-4000 kilometers) should be used to derive geophysical signals[1,4]. Ref.[1] found that monthly ocean mass changes derived from Swarm gravity fields show a root mean square error (RMSE) of 4.0 mm when compared to GRACE in the overlap period of the missions, which is comparable to or better than other Swarm solutions[5–7]. As the Swarm on-board accelerometers experience strong thermally driven drifts, strategies differ in how these problems are mitigated. Ref.[1] showed that it is essential to model the non-gravitational accelerations during their processing, while the more common strategy is to introduce additional empirical parameters. The monthly IGG-Swarm gravity field solutions contain spherical harmonic coefficients along with their formal errors. However, as shown in ref.[1], these formal errors are too optimistic. In Section *Error Budget* of the main article, we explain how the error budget for this study is derived instead.

GRACE Data

The GRACE spacecraft have been equipped with a precise K-band inter-satellite ranging system, which enabled several groups (Jet Propulsion Laboratory (JPL), Center for Space Research at University of Texas (CSR), GeoforschungsZentrum Potsdam (GFZ) and Institute of Geodesy of the Graz University of Technology (IFG), and others) to derive monthly gravity fields up to d/o 90 or more. Here, we use GRACE data for (1) deriving the spatial patterns that correspond to the dominating modes of variability in gravity and surface mass change, and (2) validating our method in the overlap period. We use all available ITSG-2018 GRACE solutions from April 2002 until June 2017[3]. Furthermore, we use ITSG-2018 GRACE-FO data for validating our results after the GRACE period. For both, GRACE and Swarm gravity fields, we consistently replaced degree 1[8] and c_{20}[9] coefficients with data related to the mentioned references (from `https://grace.jpl.nasa.gov/data/get-data/geocenter/` and `https://grace.jpl.nasa.gov/data/get-data/oblateness/`) . We then remove a mean gravity field[10] to obtain temporal anomalies, apply a correction for glacial isostatic adjustment[11] together with a 500 km Gaussian filter. The spherical harmonic coefficients are then converted to monthly grids of equivalent water height (e.w.h.)[12].

GPS Data

We use daily GPS vertical position time series processed by the Nevada Geodetic Laboratory (NGL, ref.[13]) in a PPP (Precise Point Positioning) mode (`http://geodesy.unr.edu/gps_timeseries/tenv3/`) expressed in a center-of-mass frame. NGL processes an immense number of stations located worldwide (more than 18 000 stations) which enables us to choose those relevant for regional analyses. Since our main focus is on the Swarm-based reconstruction, we use five basins and select the GPS stations whose observations almost completely cover the Swarm time span: from December 2013 until today. We then used those sets of stations for each basin and randomly selected four stations. In this way we finally ended with 20 stations total to validate our Swarm-reconstruction.

Vertical GPS displacements were pre-processed in terms of outliers and offsets. We used three times IQR value (Interquartile Range Rule) to identify and remove outlying values. Offsets were identified by their epochs, as reported in the NGL database (`http://geodesy.unr.edu/NGLStationPages/steps.txt`). Since some events are not included there, a manual inspection has been performed and additional epochs were added; their number differs for individual stations.

GPS displacements are contributed by a number of phenomena overlapping each other. As the deformation fields we estimate

are mainly induced by changes in hydrology, we remove non-tidal effects related to atmospheric and oceanic changes using geophysical fluid models processed by the Earth-System-Modelling group of Deutsches GeoForschungsZentrum (ESMGFZ; http://esmdata.gfz-potsdam.de:8080/repository). Geophysical models were re-sampled from 3-hours to daily values and interpolated from grids to stations' coordinates. Besides non-tidal effects we also removed GIA impact using the model from ref.[11], to stay consistent with GRACE processing. Finally, GPS daily vertical displacements were smoothed with a 30-day moving average filter for a better comparison.

S2 Last Months of GRACE

Towards the end of the GRACE mission, the monthly gravity fields show a lower quality. This is due to missing accelerometer data for GRACE B[14] since November 2016. GRACE A accelerometer data was used as a transplant in order to derive gravity field solutions. The last 9 monthly solutions depicted in Fig. S2.1 are evaluated up to d/o 12 and in Fig. S2.2 up to d/o 40. Missing accelerometer data clearly leads to more noise as well as regional artifacts. Antarctic regions are especially affected and show unrealistic e.w.h. values for January, March and June 2017.

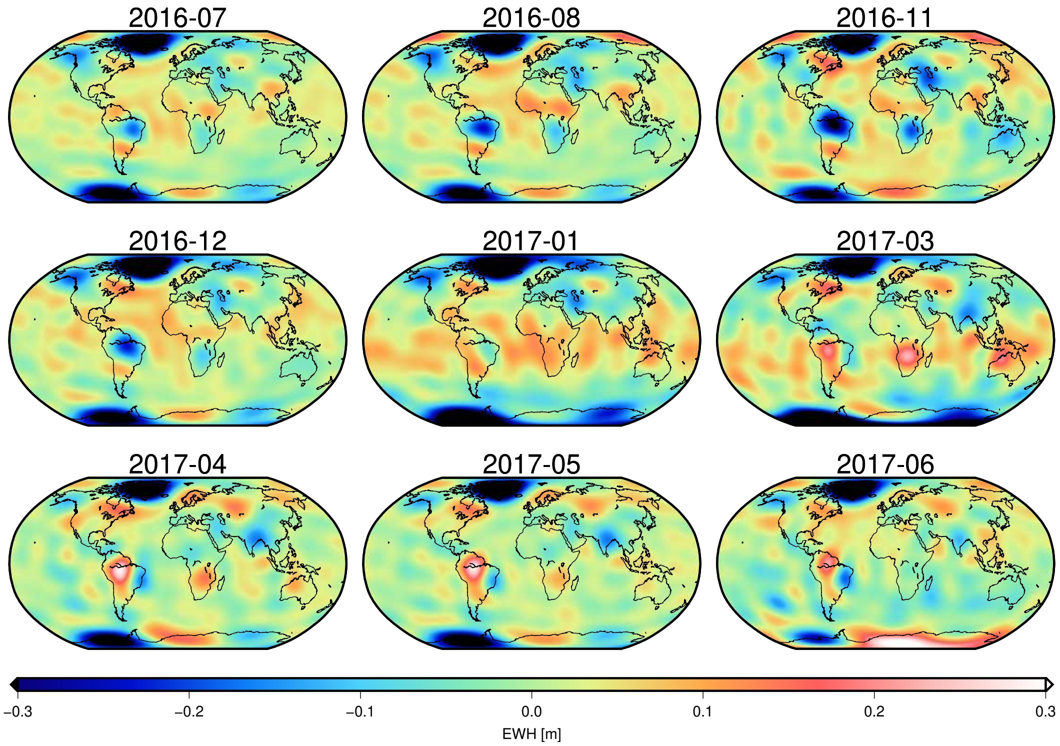

Figure S2.1. Equivalent water height of the last 9 months from GRACE, evaluated up to d/o 12.

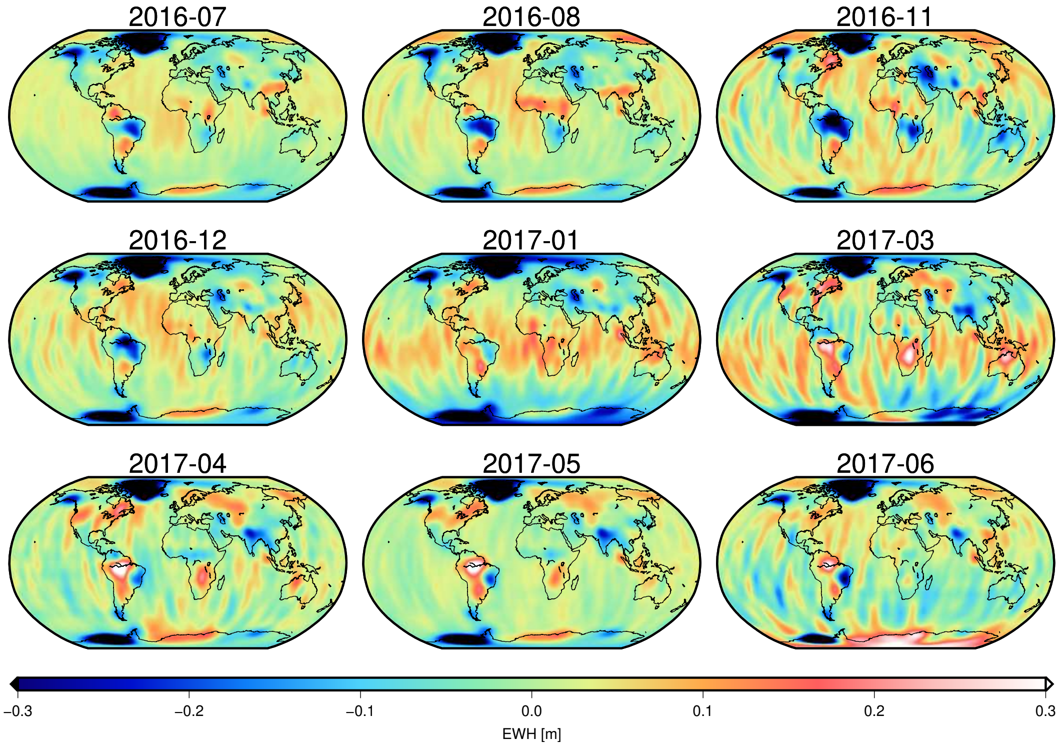

Figure S2.2. Equivalent water height of the last 9 months from GRACE, evaluated up to d/o 40.

S3 Vertical Displacements from GPS, GRACE, Monthly Swarm-only Solutions and Swarm Reconstructed Solutions

Trends that one estimates from GPS time series inevitably include, in addition to surface mass loading, a plethora of non-loading effects such as glacial isostatic adjustment, monument instability and bedrock thermal expansion. In contrast, uplift trends derived from GRACE, Swarm-only, and from the Swarm reconstructions reflect only changes in the hydrological and ice loading. Any interpretation of agreement or disagreement must thus take into account the site location and the magnitude of effects. We used two different methods to derive GPS trends and annual fits: observed daily solutions are either first averaged to monthly resolution consistent with the gravity solutions, or the daily time series are fitted directly where we employed a model described by ref[15]. We find that GPS-derived annual amplitudes are systematically underestimated by the Swarm-reconstructed displacements, with a maximum of 5 mm found for LCK4 in the Ganges basin. The largest agreement between annual amplitudes derived for GPS and Swarm-reconstructed displacements was found for Mississippi basin, showing that hydrology-driven deformation dominates for this area and significantly covers other effects that GPS is sensitive to. Vertical rates also differ to some extent, with few outliers of more than 5 $\mathrm{mm\,yr^{-1}}$. We hypothesize the cause for such differences are the multiple different stages of processing where GPS scatter is reduced, as well as the slightly differing time series models that were employed for daily and monthly resolution. These disagreements thus suggest a noise level that is inherent to the different ways of analysing GPS time series; while in the following we will refer only to results from the first approach, this must be kept in mind when referring to literature GPS estimates.

Greenland and West Antarctica are affected by present-day ice melting of the ice sheets and GPS suggests a significant uplift of 1 $\mathrm{mm\,yr^{-1}}$–16 $\mathrm{mm\,yr^{-1}}$, depending on the location. For East Antarctica, only one GPS location (SYOG) has been selected, which subsides by (0.5 ± 0.1) $\mathrm{mm\,yr^{-1}}$. For SYOG, WHTM (Antarctica) and GROK (Greenland), the differences between trends estimated from GPS and Swarm-reconstructed displacements are below 1 $\mathrm{mm\,yr^{-1}}$ and GRACE and Swarm-reconstructions are close. For the remaining stations, differences are larger – this can be observed for the Ganges basin with the largest difference of 9.5 $\mathrm{mm\,yr^{-1}}$ for CHLM station, where present-day tectonic uplift of the Nepal Himalaya masks hydrology loading. In contrast, for stations in river deltas, far away from tectonically active areas, the trends derived for Swarm-reconstructed and GPS displacements are expected to agree much more closely, while discrepancies may be present due to sediment compaction or anthropogenic influences. In fact, for the Amazon and Mississippi basins, the differences in trend between GPS and Swarm-reconstructed displacements are significantly smaller as compared to other basins. Differences between GRACE and the different Swarm solutions correspond to what we have seen when discussing mass change in basins. Trends from GPS and Swarm-reconstructed solutions agree within 1 $\mathrm{mm\,yr^{-1}}$ for five (OKBF and TN46 in Mississippi basin, WHTM and SYOG in Antarctica, SNDL in Ganges basin) out of twenty stations, whereas for only eight stations, trends are in large disagreement (i.e. larger than 3 $\mathrm{mm\,yr^{-1}}$, with a change in sign). We suggest that the LCK4 time series is too short for assessing trend differences. There is a change in trend in 2012 seen in GPS displacements at HJOR; from 2012 onwards both GPS and Swarm-reconstructed displacements match each other.

Both Swarm-reconstructed and GRACE-predicted displacements reproduce well the inter-annual signals observed by GPS. Inter-annual variations are large for the Amazon basin with an evident jump in 2015, which can be noticed for all GPS locations. This is covered well by GRACE and Swarm-reconstructions. Also the 2015-2016 Amazon drought, which was related to a strong El Niño, caused an abrupt uplift of the crust which could be observed consistently by GPS, GRACE and Swarm. Inter-annual signals that were observed in GRACE and Swarm-reconstructions for three sites (MOKV, OKBF and TN46) in the Mississippi basin are also well-detected by GPS.

At the annual timescale, the vertical motion as observed by GPS is underestimated by 1 mm–5 mm for almost all stations when derived from the Swarm-reconstructed gravity solutions (Figs. 4 of the main article and S3.1-S3.5 of the Supplement). All Swarm solutions inevitably lack spatial resolution and we suspect that in addition, other effects such as thermal expansion of bedrock, GPS-specific errors, or mismodelling of non-tidal atmospheric or ocean loading mask true loading signals observed by individual stations. Best agreement between GPS and Swarm was found for sites in the Mississippi basin, where hydrologically driven deformation dominates. The timing of the annual oscillation matches each other well for GPS and Swarm-reconstructed displacements for all basins that we discuss (not shown in the table). However, we conclude that in our GPS validation experiment, at the annual timescale no clear picture emerges. Gravity solutions from the two variants of the Swarm reconstruction approach seem to outperform the monthly Swarm-only solution for some regions with large signal, i.e. for 30 % of stations, while for others the Swarm-monthly solutions appear closer to GPS. By construction the reconstruction method when applied in remove-restore mode will provide fits very close to the GRACE-predicted loading and thus performs well where we know from other studies that GRACE fits well to GPS – these are Amazon and Mississippi basins. Yet neither Swarm-only nor Swarm reconstructed solutions appear to fit GPS worse.

Antarctica						
Station	GPS (daily)	GPS (monthly)	GRACE	Swarm-only	Swarm-rec	Swarm-rec$_{residual}$
THU4	-2.4 ± 0.3	-2.6 ± 0.1	2.4 ± 0.1	1.6 ± 0.1	1.3 ± 0.1	2.5 ± 0.1
SUGG	3.8 ± 0.3	3.2 ± 0.1	2.0 ± 0.1	1.8 ± 0.1	1.1 ± 0.1	2.0 ± 0.1
WHTM	5.3 ± 1.8	1.5 ± 0.1	2.3 ± 0.1	1.7 ± 0.1	1.3 ± 0.1	2.3 ± 0.1
SYOG	-0.1 ± 0.1	-0.5 ± 0.1	-0.2 ± 0.1	0.8 ± 0.1	-0.6 ± 0.1	-0.2 ± 0.1
Amazon						
Station	GPS (daily)	GPS (monthly)	GRACE	Swarm-only	Swarm-rec	Swarm-rec$_{residual}$
AMCO	0.1 ± 1.4	1.2 ± 0.1	-0.4 ± 0.1	2.6 ± 0.1	1.8 ± 0.1	-0.4 ± 0.1
NAUS	-1.6 ± 2.2	-1.2 ± 0.1	-0.4 ± 0.1	2.4 ± 0.1	2.2 ± 0.1	-0.4 ± 0.1
APLJ	1.6 ± 1.6	1.0 ± 0.1	-0.4 ± 0.1	2.1 ± 0.1	2.0 ± 0.1	-0.4 ± 0.1
PAIT	1.6 ± 1.6	2.1 ± 0.1	-0.3 ± 0.1	2.4 ± 0.1	2.4 ± 0.1	-0.4 ± 0.1
Mississippi						
Station	GPS (daily)	GPS (monthly)	GRACE	Swarm-only	Swarm-rec	Swarm-rec$_{residual}$
OKBF	0.1 ± 0.2	0.5 ± 0.1	0.1 ± 0.1	-0.4 ± 0.1	0.0 ± 0.1	0.1 ± 0.1
MOKV	-1.3 ± 0.3	-1.3 ± 0.1	0.1 ± 0.1	0.3 ± 0.1	0.1 ± 0.1	0.1 ± 0.1
TN46	-0.1 ± 0.4	0.0 ± 0.1	-0.1 ± 0.1	0.2 ± 0.1	-0.1 ± 0.1	-0.1 ± 0.1
KYTI	-2.9 ± 0.3	-2.7 ± 0.1	-0.1 ± 0.1	0.7 ± 0.1	-0.1 ± 0.1	-0.1 ± 0.1
Greenland						
Station	GPS (daily)	GPS (monthl)	GRACE	Swarm-only	Swarm-rec	Swarm-rec$_{residual}$
GROK	8.4 ± 1.1	2.9 ± 0.1	2.3 ± 0.1	0.9 ± 0.1	1.8 ± 0.1	2.3 ± 0.1
HEL2	15.2 ± 2.3	15.9 ± 0.1	2.8 ± 0.1	3.4 ± 0.1	2.1 ± 0.1	2.8 ± 0.1
HJOR	6.2 ± 1.9	5.1 ± 0.1	2.5 ± 0.1	3.3 ± 0.1	1.8 ± 0.1	2.5 ± 0.1
SENU	8.1 ± 1.4	6.8 ± 0.1	2.1 ± 0.1	2.8 ± 0.1	1.6 ± 0.1	2.1 ± 0.1
Ganges						
Station	GPS (daily)	GPS (monthly)	GRACE	Swarm-only	Swarm-rec	Swarm-rec$_{residual}$
LCK4	-1.7 ± 0.3	-2.3 ± 0.1	0.2 ± 0.1	-0.9 ± 0.1	-0.2 ± 0.1	0.2 ± 0.1
BRN2	-1.2 ± 0.2	-1.6 ± 0.1	0.1 ± 0.1	-2.0 ± 0.1	-0.3 ± 0.1	0.1 ± 0.1
SNDL	0.5 ± 0.1	0.2 ± 0.1	0.2 ± 0.1	-1.6 ± 0.1	-0.2 ± 0.1	0.2 ± 0.1
CHLM	9.5 ± 0.2	9.3 ± 0.1	0.2 ± 0.1	-1.5 ± 0.1	-0.2 ± 0.1	0.2 ± 0.1

Table S3.1. Vertical trends and their errors (given in $mm\,yr^{-1}$) estimated for GPS vertical displacements, and vertical displacements estimated from GRACE observations, Swarm-only observations, Swarm-reconstructed and Swarm-reconstructed$_{residual}$ data. For the GPS daily displacements, we assumed a time series model, as described in[15] with a combination of white and power-law noises assumed during the estimates with the Maximum Likelihood Estimation[16]. For the GPS monthly displacements and displacements based on GRACE, Swarm-only, Swarm-reconstructed and Swarm-reconstructed$_{residual}$ data, we assumed trend, annual and semi-annual signals. For the stochastic part, a white noise-only has been assumed, since time series are too short to account for the power-law noise.

Figures S3.1-S3.5 show the remaining time series of vertical displacements that were not shown in Fig. 4 of the main article. Furthermore, Tables S3.2 and S3.1 summarize all amplitudes and trends.

Antarctica												
Station	GPS (daily)		GPS (monthly)		GRACE		Swarm-only		Swarm-rec		$Swarm\text{-}rec_{residual}$	
THU4	6.3	± 0.8	4.5	± 0.1	0.3	± 0.1	2.2	± 0.1	1.3	± 0.1	0.3	± 0.1
SUGG	5.1	± 0.8	2.9	± 0.1	0.6	± 0.1	1.5	± 0.1	1.3	± 0.1	0.6	± 0.1
WHTM	8.2	± 1.7	4.4	± 0.1	0.6	± 0.1	1.7	± 0.1	1.4	± 0.1	0.6	± 0.1
SYOG	2.5	± 0.3	2.1	± 0.1	0.4	± 0.1	2.0	± 0.1	1.5	± 0.1	0.3	± 0.1
Amazon												
Station	GPS (daily)		GPS (monthly)		GRACE		Swarm-only		Swarm-rec		$Swarm\text{-}rec_{residual}$	
AMCO	11.3	± 1.2	11.4	± 0.1	10.5	± 0.1	9.9	± 0.1	7.5	± 0.1	10.5	± 0.1
NAUS	27.8	± 0.9	27.0	± 0.1	11.7	± 0.1	9.9	± 0.1	8.0	± 0.1	11.7	± 0.1
APLJ	12.1	± 2.3	13.4	± 0.1	10.6	± 0.1	8.5	± 0.1	6.9	± 0.1	10.6	± 0.1
PAIT	16.5	± 1.2	16.8	± 0.1	12.4	± 0.1	10.5	± 0.1	9.1	± 0.1	12.4	± 0.1
Mississippi												
Station	GPS (daily)		GPS (monthly)		GRACE		Swarm-only		Swarm-rec		$Swarm\text{-}rec_{residual}$	
OKBF	1.8	± 0.4	1.8	± 0.1	1.7	± 0.1	2.5	± 0.1	1.6	± 0.1	1.7	± 0.1
MOKV	3.2	± 0.6	1.9	± 0.1	2.1	± 0.1	1.8	± 0.1	2.0	± 0.1	2.1	± 0.1
TN46	4.9	± 0.7	3.8	± 0.1	1.4	± 0.1	1.5	± 0.1	1.3	± 0.1	1.5	± 0.1
KYTI	2.3	± 0.5	3.6	± 0.1	1.6	± 0.1	1.3	± 0.1	1.5	± 0.1	1.6	± 0.1
Greenland												
Station	GPS (daily)		GPS (monthl)		GRACE		Swarm-only		Swarm-rec		$Swarm\text{-}rec_{residual}$	
GROK	2.4	± 1.2	1.1	± 0.1	0.8	± 0.1	0.8	± 0.1	1.5	± 0.1	0.8	± 0.1
HEL2	5.4	± 2.1	4.8	± 0.1	1.2	± 0.1	1.9	± 0.1	2.0	± 0.1	1.2	± 0.1
HJOR	5.0	± 1.9	4.6	± 0.1	1.1	± 0.1	2.6	± 0.1	1.9	± 0.1	1.2	± 0.1
SENU	6.0	± 1.8	6.0	± 0.1	1.2	± 0.1	3.3	± 0.1	1.8	± 0.1	1.2	± 0.1
Ganges												
Station	GPS (daily)		GPS (monthly)		GRACE		Swarm-only		Swarm-rec		$Swarm\text{-}rec_{residual}$	
LCK4	12.8	± 1.9	7.8	± 0.1	4.6	± 0.1	6.5	± 0.1	2.8	± 0.1	4.6	± 0.1
BRN2	2.6	± 0.7	3.9	± 0.1	5.9	± 0.1	7.5	± 0.1	4.2	± 0.1	5.9	± 0.1
SNDL	9.9	± 0.6	10.2	± 0.1	5.4	± 0.1	7.2	± 0.1	3.7	± 0.1	5.4	± 0.1
CHLM	10.0	± 0.5	9.9	± 0.1	5.1	± 0.1	7.1	± 0.1	3.4	± 0.1	5.1	± 0.1

Table S3.2. Annual amplitudes and their errors (in mm) estimated for GPS vertical displacements, and vertical displacements estimated from GRACE observations, Swarm-only observations, Swarm-reconstructed and $Swarm\text{-}reconstructed_{residual}$ data. A time series model has been adopted as described above for Tab. S3.1.

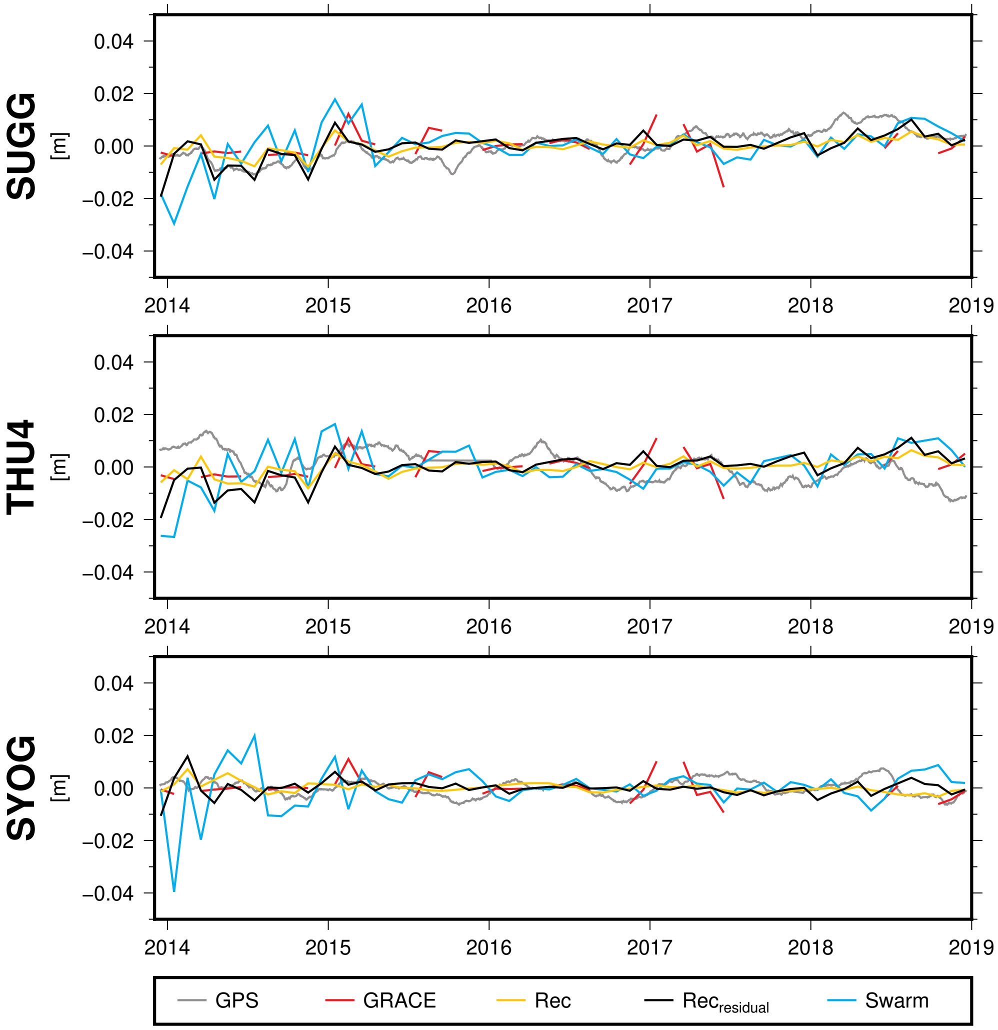

Figure S3.1. Daily vertical displacements derived for GPS stations: SUGG, THU4, WHTM and SYOG in Antarctica, plotted in gray. These were smoothed with a 30-day moving average filter for a better visibility. Vertical displacements were also retrieved for GPS locations from GRACE observations, Swarm reconstructed, Swarm reconstructed$_{residual}$ and Swarm-only data and plotted in red, yellow, black and blue, respectively.

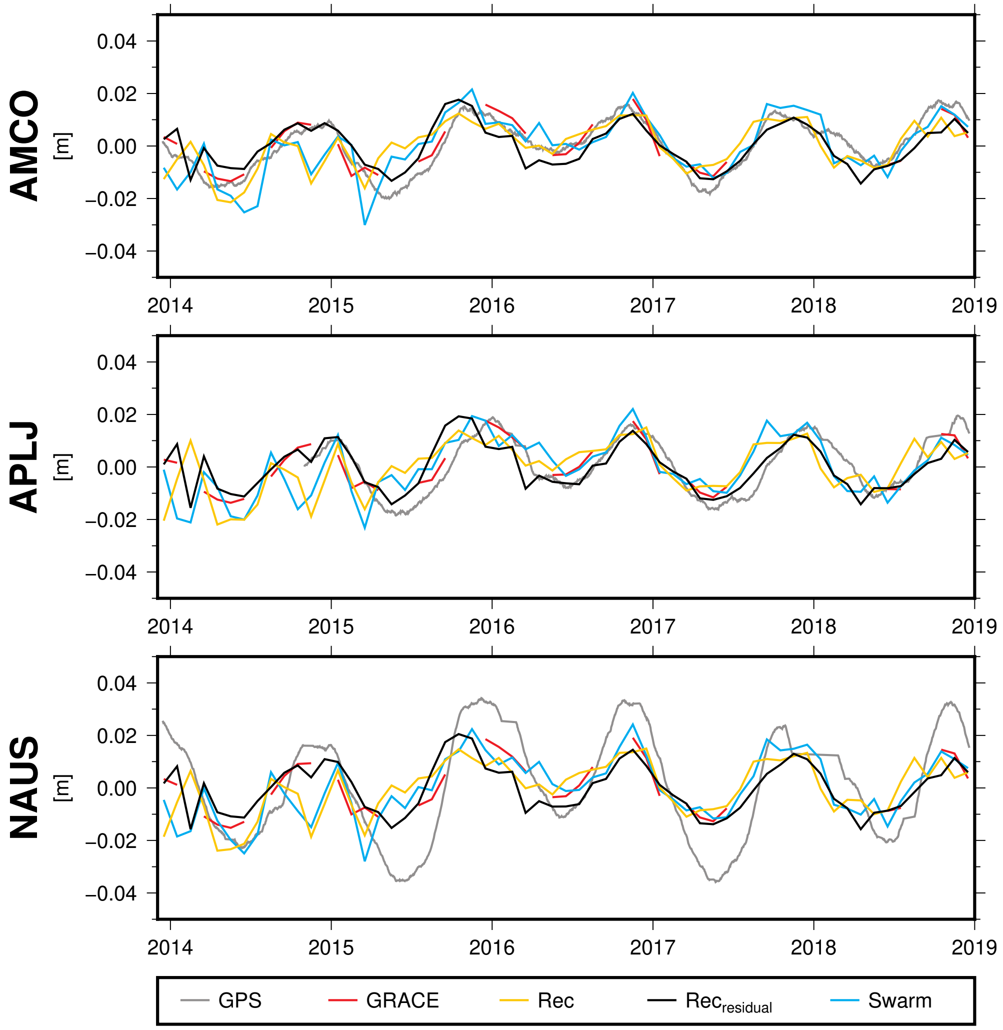

Figure S3.2. Daily vertical displacements derived for GPS stations: AMCO, APLJ, PAIT, NAUS in the Amazon basin, plotted in gray. These were smoothed with a 30-day moving average filter for a better visibility. Vertical displacements were also retrieved for GPS locations from GRACE observations, Swarm reconstructed, Swarm reconstructed$_{\text{residual}}$ and Swarm-only data and plotted in red, yellow, black and blue, respectively.

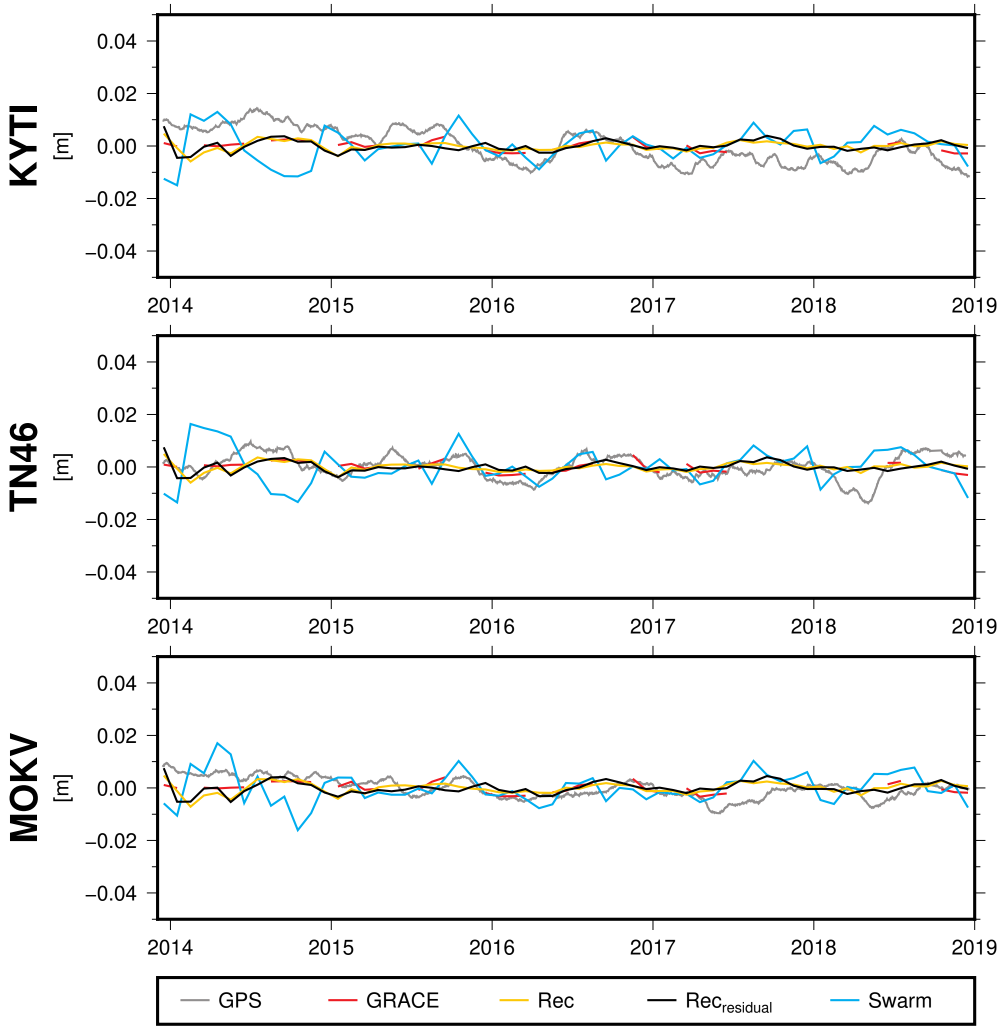

Figure S3.3. Daily vertical displacements derived for GPS stations: KYTI, OKBF, TN46, MOKV in the Mississippi basin, plotted in gray. These were smoothed with a 30-day moving average filter for a better visibility. Vertical displacements were also retrieved for GPS locations from GRACE observations, Swarm reconstructed, Swarm reconstructed$_{residual}$ and Swarm-only data and plotted in red, yellow, black and blue, respectively.

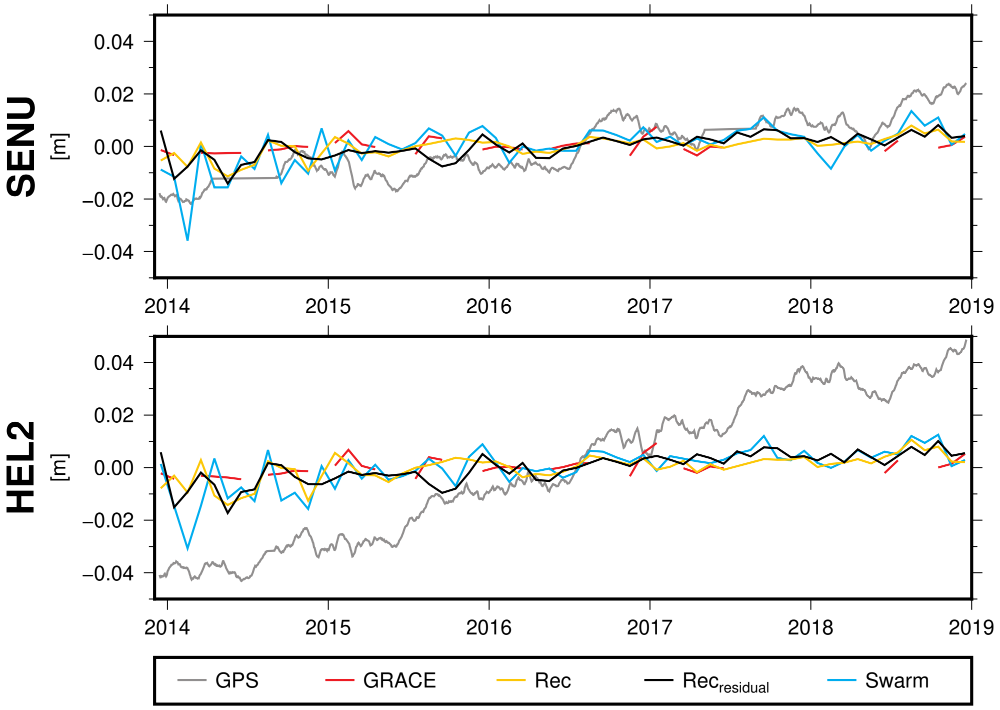

Figure S3.4. Daily vertical displacements derived for GPS stations: SENU and HEL2 in Greenland, plotted in gray. These were smoothed with a 30-day moving average filter for a better visibility. Vertical displacements were also retrieved for GPS locations from GRACE observations, Swarm reconstructed, Swarm reconstructed$_{\mathrm{residual}}$ and Swarm-only data and plotted in red, yellow, black and blue, respectively.

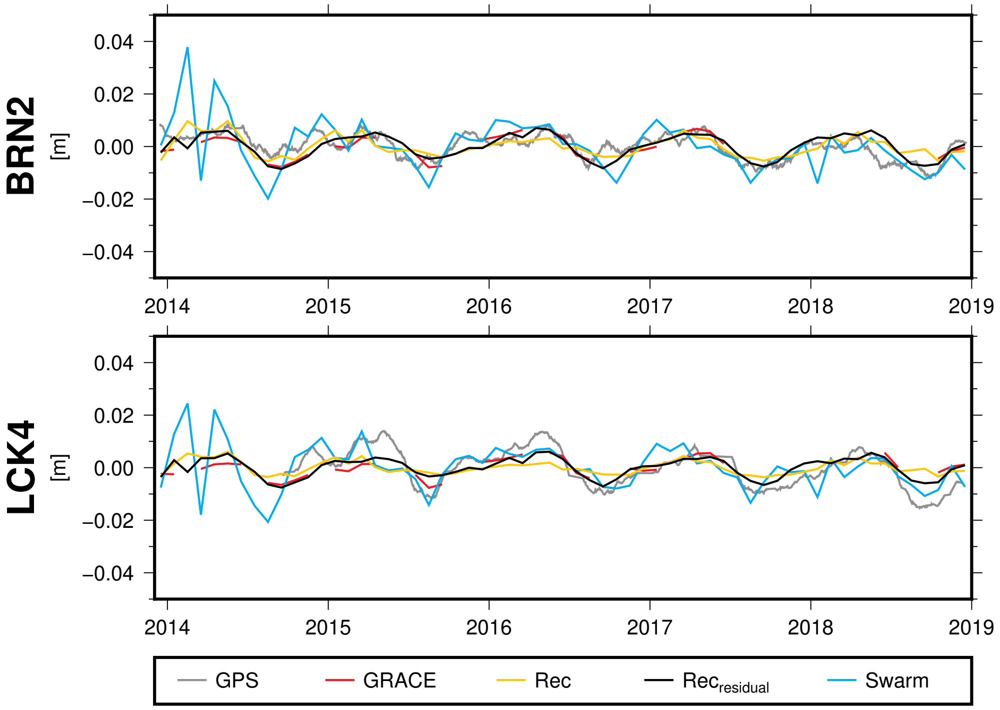

Figure S3.5. Daily vertical displacements derived for GPS stations: BRN2, LCK4, CHLM, SNDL in the Ganges basin, plotted in light gray. plotted in gray. These were smoothed with a 30-day moving average filter for a better visibility. Vertical displacements were also retrieved for GPS locations from GRACE observations, Swarm reconstructed, Swarm reconstructed$_{\mathrm{residual}}$ and Swarm-only data and plotted in red, yellow, black and blue, respectively.

S4 Comparison of monthly Swarm Reconstruction to GRACE 6-Parameter Signal Model

As mentioned in Section "Basin Averages", a common approach to bridge the GRACE gaps is to inter- or extrapolate previous and subsequent GRACE solutions using a simple six-parameter model (constant, trend, annual and semiannual terms). This works well, as long as mass changes evolve regularly over time. However, a simple interpolation would not be able to detect larger interannual variabilities, which deviate from a "regular" behaviour. On the basis of comparisons to GRACE, our findings are that monthly Swarm reconstructions should be generally preferred to a simple six-parameter GRACE model. Instead on relying on a single snapshot only, we artificially introduce an 11-months gap, corresponding to the real gap between GRACE and GRACE-FO, and subsequently shift it through the GRACE/Swarm overlapping period. The remaining GRACE data is then used to fill the gap by fitting the six-parameter model and using the real GRACE data as the ground-truth, we compute RMSE values from the model as well as from monthly and reconstructed Swarm solutions. We conclude (Fig. S4.1 for d/o 12 and Fig. S4.2 for d/o 40) that the reconstruction method indeed always (except for the Amazon basin) turns out as a more reliable gap filler than the monthly Swarm-only solution. For a conservative error estimate for the six-parameter model extrapolation, we turn to the RMSE associated with the largest temporal anomaly within the entire GRACE time period - we argue that for any future mission data gap we cannot know the degree of temporal anomaly. This can be debated, but we note that the approximation error of any extrapolation method mathematically must rely of assumptions of smoothness which are hard to come by. This having been said, in general the monthly Swarm reconstructions lead to a lower RMSE w.r.t. GRACE, compared to the 6-parameter model. Larger errors are found only in the early Swarm period for the Greenland, inner Europe and the Ganges and Danube basins. This can be explained by the lower quality of Swarm at the beginning of the mission and the error of Swarm-only and the Swarm reconstruction in 2016/2017 should rather be taken into account as an estimate for the real GRACE/GRACE-FO data gap. The Swarm reconstruction$_{\text{residual}}$ variant mostly leads to changes in the early Swarm period for Fig. S4.1 and S4.2. To conclude, in the real data gaps, we suggest that the new monthly Swarm reconstruction may be preferred to the monthly Swarm-only solution or simply interpolating the existing GRACE solutions.

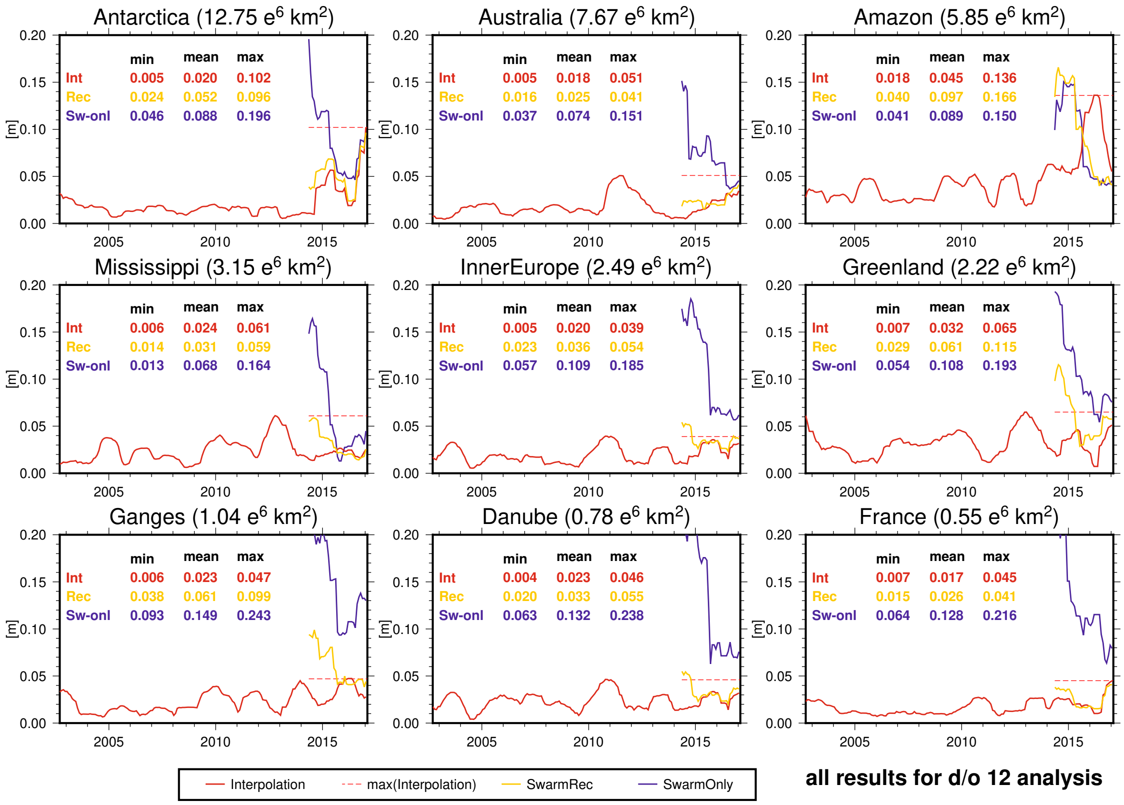

Figure S4.1. Results of three gap-filler methods for nine different regions for d/o 12. Red: An artificial gap of 11 months is assumed in the GRACE time series of equivalent water height for each respective region. Then, data in this gap is interpolated from the remaining GRACE data (constant term, trend, annual, semiannual). An RMS value [m] of (GRACE-true vs. GRACE-interpolated) is computed for this gap and plotted in red. The artifical gap is shifted through the whole GRACE period and the RMS is always plotted for the mid-point of each 11-months gap. The maximum RMS is the error that we assume for the interpolation method. It is marked with the dashed-red line. Yellow: RMS [m] of (Swarm reconstruction vs. GRACE). The RMS is computed in 11-months intervals and plotted in yellow for the mid-point of each interval. Purple: RMS [m] of (Swarm-only vs. GRACE). The RMS is computed in 11-months intervals and plotted in purple for the mid-point of each interval.

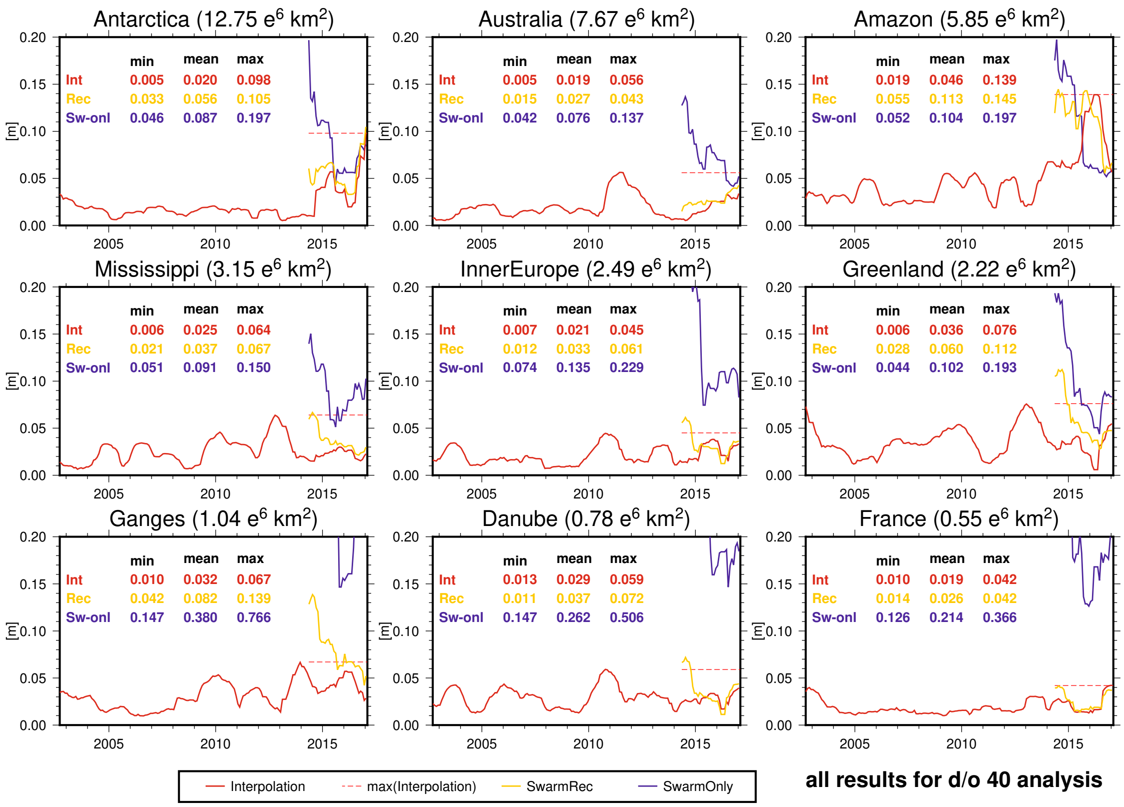

Figure S4.2. Results of three gap-filler methods for nine different regions for d/o 40. Red: An artificial gap of 11 months is assumed in the GRACE time series of equivalent water height for each respective region. Then, data in this gap is interpolated from the remaining GRACE data (constant term, trend, annual, semiannual). An RMS value [m] of (GRACE-true vs. GRACE-interpolated) is computed for this gap and plotted in red. The artifical gap is shifted through the whole GRACE period and the RMS is always plotted for the mid-point of each 11-months gap. The maximum RMS is the error that we assume for the interpolation method. It is marked with the dashed-red line. Yellow: RMS [m] of (Swarm reconstruction vs. GRACE). The RMS is computed in 11-months intervals and plotted in yellow for the mid-point of each interval. Purple: RMS [m] of (Swarm-only vs. GRACE). The RMS is computed in 11-months intervals and plotted in purple for the mid-point of each interval.

S5 Choice of three EOFs

The choice of three EOFs is a result of a global analysis of the RMS of the Swarm reconstruction w.r.t GRACE and a regional analysis of basin averages of our study regions and additional further regions (see Fig. S5.1 for d/o 12 and Fig. S5.2 for d/o 40). In Figs. S5.1 and S5.2 we compute time series of equivalent water height for each basin and compared four cases of RMS values as a function of the number of employed EOFs. For each case, we computed the RMS of the respective time series w.r.t. GRACE for 2 – 30 EOFs. The following cases were investigated: (1) GRACE PCA with 2 – 30 EOFs vs. the full GRACE signal (red). This shows us, if we are able to see the mass changes in each region if we use a truncated version of the GRACE PCA, (2) Swarm Reconstruction with 2 – 30 EOFs vs. the full GRACE signal (yellow). This shows us basically two effects: the error due to the lower resolution of Swarm w.r.t. GRACE and the error due to the truncation, (3) Swarm Reconstruction with 2 – 30 EOFs vs GRACE PCA with x EOFs (blue). Here, only the error due to the lower quality of Swarm w.r.t. GRACE is depicted, as we apply the same truncation for both time series, (4) Swarm-only vs. the full GRACE signal (purple). This is independent from the number of chosen EOFs, as we compare the original time series (without applying PCA) to each other.

The Swarm reconstruction is always (except for the Amazon basin) better than the monthly Swarm-only solution (case (2) vs. case (4)). For a high number of EOFs (>15) cases (2) and (3) are always very close to each other and often converge towards case (4). This means that the error is then mainly due to the lower quality of Swarm compared to GRACE and the error due to the truncation is negligible. For <10 EOFs there is a difference between cases (2) and (3), which means that the error due to truncation does also have an effect on the solution. For a perfect solution, case (2) would equal zero. This is, of course, not possible, as we have two sources of errors: the lower quality of Swarm and the truncation effect. Thus, we should get case (3) as low as possible, then we minimize noise due to the Swarm-only solution in the Swarm reconstructed solution. The results confirm that for all investigated regions, the choice of 3 EOFs leads to good (for most regions: the best) results.

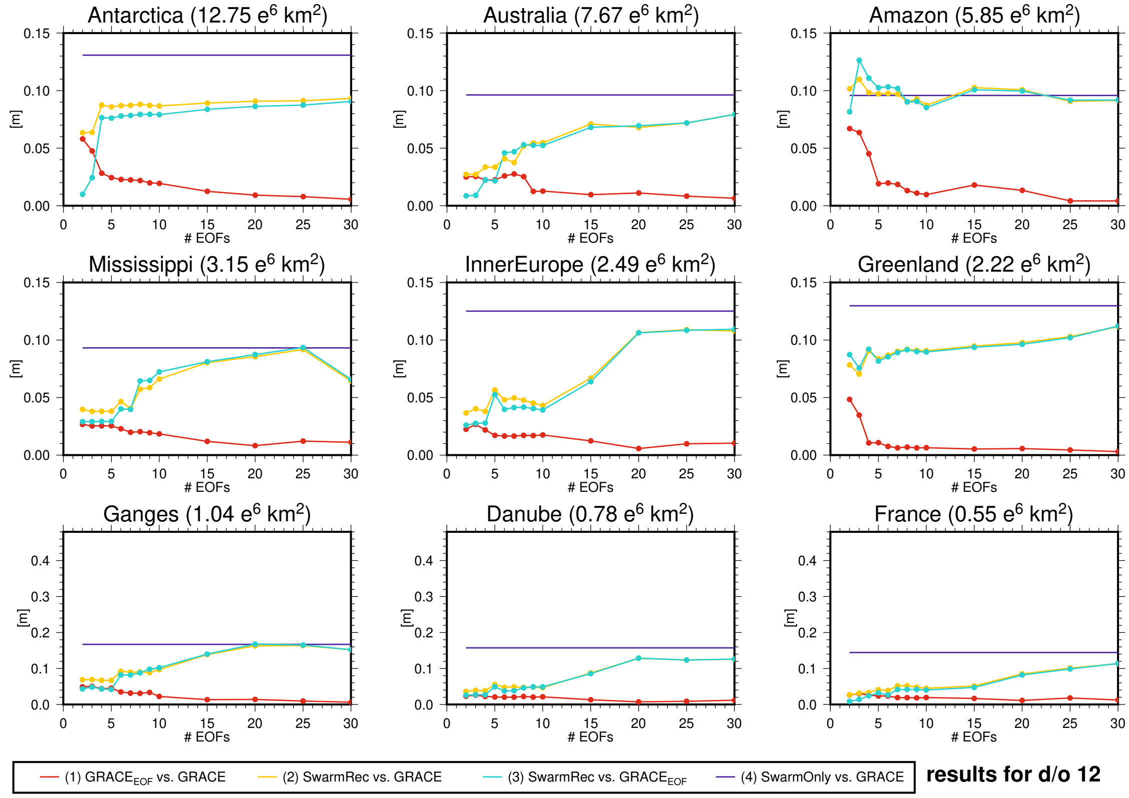

Figure S5.1. RMSE values [m of equivalent water height] of basin averages from 2013-12 to 2017-06 with respect to GRACE for nine different regions. The results are plotted against the number of employed EOFs for d/o 12. Red: GRACE PCA with X EOFs against the full GRACE signal. Yellow: Swarm Reconstruction vs. the full GRACE signal. Blue: Swarm Reconstruction against the GRACE PCA with X EOFs. Purple: Swarm-only vs. the full GRACE signal (independent of the number of EOFs).

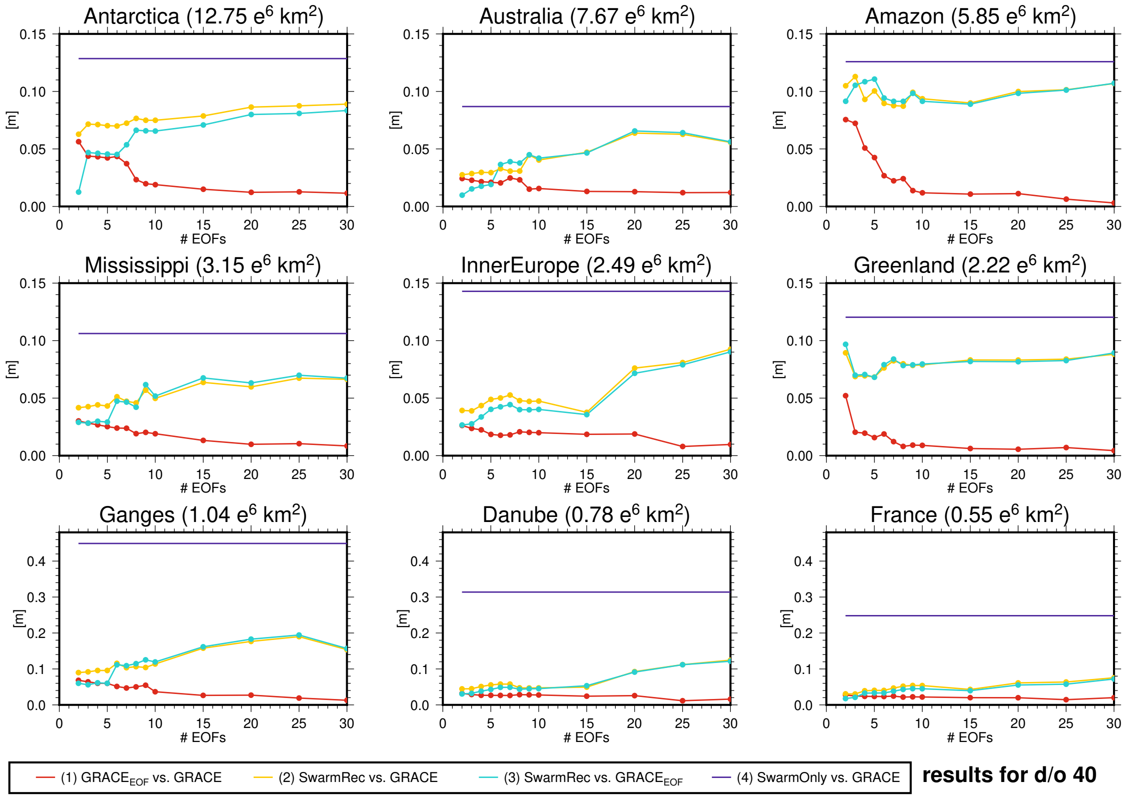

Figure S5.2. RMSE values [m of equivalent water height] of basin averages from 2013-12 to 2017-06 with respect to GRACE for nine different regions. The results are plotted against the number of employed EOFs for d/o 40. Red: GRACE PCA with X EOFs against the full GRACE signal. Yellow: Swarm Reconstruction vs. the full GRACE signal. Blue: Swarm Reconstruction against the GRACE PCA with X EOFs. Purple: Swarm-only vs. the full GRACE signal (independent of the number of EOFs).

References

1. Lück, C., Kusche, J., Rietbroek, R. & Löcher, A. Time-variable gravity fields and ocean mass change from 37 months of kinematic Swarm orbits. *Solid Earth* **9**, 323–339, DOI: 10.5194/se-9-323-2018 (2018).

2. Mayer-Gürr, T. *Gravitationsfeldbestimmung aus der Analyse kurzer Bahnbögen am Beispiel der Satellitenmissionen CHAMP und GRACE.* Dissertation, Universität Bonn (2006).

3. Kvas, A. *et al.* ITSG-Grace2018: Overview and Evaluation of a New GRACE-Only Gravity Field Time Series. *J. Geophys. Res. Solid Earth* **124**, 9332–9344, DOI: 10.1029/2019JB017415 (2019).

4. Encarnação De Teixeira, J. *et al.* Description of the multi-approach gravity field models from Swarm GPS data. *Earth System Science Data* **12**, 1385–1417, DOI: 10.5194/essd-12-1385-2020 (2020).

5. Jäggi, A. *et al.* Swarm kinematic orbits and gravity fields from 18 months of GPS data. *Adv. Space Res.* **57**, 218 – 233, DOI: http://dx.doi.org/10.1016/j.asr.2015.10.035 (2016).

6. Bezděk, A., Sebera, J., Teixeira da Encarnação, J. & Klokočník, J. Time-variable gravity fields derived from GPS tracking of Swarm. *Geophys. J. Int.* **205**, 1665–1669, DOI: 10.1093/gji/ggw094 (2016).

7. Zehentner, N. & Mayer-Gürr, T. Precise orbit determination based on raw GPS measurements. *J. Geod.* **90**, 275–286, DOI: 10.1007/s00190-015-0872-7 (2016).

8. Cheng, M., Tapley, B. D. & Ries, J. C. Deceleration in the Earth's oblateness. *J. Geophys. Res. Solid Earth* **118**, 740–747, DOI: 10.1002/jgrb.50058 (2013).

9. Swenson, S., Chambers, D. & Wahr, J. Estimating geocenter variations from a combination of GRACE and ocean model output. *J. Geophys. Res. Solid Earth* **113**, DOI: 10.1029/2007JB005338 (2008). B08410.

10. Pail, R., Gruber, T., Fecher, T. & GOCO Project Team. The Combined Gravity Model GOCO05c. GFZ Data Services, DOI: http://doi.org/10.5880/icgem.2016.003 (2016).

11. A, G., Wahr, J. & Zhong, S. Computations of the viscoelastic response of a 3-D compressible Earth to surface loading: an application to Glacial Isostatic Adjustment in Antarctica and Canada. *Geophys. J. Int.* **192**, 557–572, DOI: 10.1093/gji/ggs030 (2013).

12. Wahr, J., Molenaar, M. & Bryan, F. Time variability of the Earth's gravity field: Hydrological and oceanic effects and their possible detection using GRACE. *J. Geophys. Res.* **103**, 30205–30230 (1998).

13. Blewitt, G., Hammond, W. & Kreemer, C. Harnessing the gps data explosion for interdisciplinary science. *Eos* **99**, DOI: 10.1029/2018EO104623 (2018).

14. Flechtner, F., Bettadpur, S., Gerhard, K. & Christoph, D. GRACE Science Data System Monthly Report. Tech. Rep., GFZ (2017).

15. Bogusz, J. & Klos, A. On the Significance of Periodic Signals in Noise Analysis of GPS Station Coordinates Time Series. *GPS Solutions* **20**, 655–664, DOI: 10.1007/s10291-015-0478-9 (2016).

16. Langbein, J. Estimating rate uncertainty with maximum likelihood: differences between power-law and flicker–random-walk models. *J. Geod.* **86**, 775–783, DOI: 10.1007/s00190-012-0556-5 (2012).
